# Effects of Dietary Glucose, Fructose, and Monosaccharide-to-Lard Energy Ratios on Cecal Microbiota Composition and Ecological Organization in Rats

**DOI:** 10.3390/nu18172875

**Published:** 2026-09-02

**Authors:** József Szabó, Gergely Maróti, Norbert Solymosi, Emese Andrásofszky, Tamás Tuboly, András Bersényi, Geza Bruckner, Hedvig Fébel

**Affiliations:** 1 Institute for Animal Breeding, Nutrition and Laboratory Animal Science, University of Veterinary Medicine, P.O. Box 2, H-1400 Budapest, Hungary; andrasofszky.emese@univet.hu (E.A.); bersenyi.andras@univet.hu (A.B.); 2 Institute of Plant Biology, Hun-Ren Biological Research Center, H-6726 Szeged, Hungary; marotig@seqomics.hu; 3 Centre for Bioinformatics, University of Veterinary Medicine, P.O. Box 2, H-1400 Budapest, Hungary; solymosi.norbert@gmail.com; 4 Department of Microbiology and Infectious Diseases, University of Veterinary Medicine, P.O. Box 2, H-1400 Budapest, Hungary; 5 Department of Athletic Training and Clinical Nutrition, University of Kentucky, Lexington, KY 40536, USA; gezabruckner0@gmail.com; 6 Department of Obstetrics and Food Animal Medicine Clinic, University of Veterinary Medicine, P.O. Box 2, H-1400 Budapest, Hungary; febel.hedvig@univet.hu

**Keywords:** gut microbiota, dietary fat, glucose, fructose, monosaccharides, microbial ecology, Bacteroidota, Bacillota, humoral immune response, lard

## Abstract

**Background:** Although dietary fat and carbohydrates are major determinants of gut microbiota composition, their interactive effects across changing dietary monosaccharide-to-lard energy ratios remain incompletely understood. This study descriptively examined treatment-level cecal microbiome profiles across dietary gradients in which lard (L) was progressively replaced with glucose (G) or fructose (F). **Methods:** A carbohydrate-free, lard-rich control diet was formulated, and lard was progressively replaced with glucose or fructose while maintaining a constant protein-to-energy ratio. Cecal contents from eight rats per dietary group were pooled in equal amounts, yielding one composite microbiome sample per treatment. Pooled samples were characterized by shotgun metagenomic sequencing. Sequencing/classified read counts (CRs) and relative abundance (RA) were treated as complementary sequencing-derived representations rather than measures of absolute bacterial abundance. Microbiome outcomes were interpreted descriptively at the treatment level. **Results:** Across the pooled treatment profiles, CRs and RA showed non-linear patterns and did not consistently change in parallel, providing complementary descriptions of treatment-level taxonomic responses. CR patterns indicated a combined effect of L and monosaccharide content, with several mixed L–monosaccharide diets showing lower CRs than both the L_6.03_ reference and the lard-free endpoints. Differences between the G and F series were most apparent at low L and high monosaccharide levels, particularly under lard-free conditions, although their magnitude and direction varied among taxa. Hierarchical clustering and exploratory correlation networks provided complementary descriptions of treatment-level community organization. **Conclusions:** The pooled treatment-level microbiome profiles revealed non-linear responses to changing dietary L–monosaccharide composition, with CRs and RA providing partly different information on taxonomic patterns. Differences between the G and F series were most apparent at low L and high monosaccharide levels, particularly under lard-free conditions.

## 1. Introduction

Diet is one of the principal determinants of gut microbiota composition, diversity, and function. Long-term dietary patterns differing in fat and carbohydrate composition have been associated with distinct microbial communities and gastrointestinal disease, including colorectal cancer [1,2]. Specific dietary components may contribute differently to metabolic outcomes, including metabolic-dysfunction-associated fatty liver disease (MAFLD): diets rich in saturated fat and refined sugars, particularly fructose-containing sugars, have been associated with hepatic lipid accumulation and adverse metabolic profiles, whereas fiber-, unsaturated-fatty-acid-, and plant-rich dietary patterns, such as the Mediterranean diet, have been associated with more favorable metabolic and hepatic outcomes [3]. Fiber-rich plant foods also provide fermentable substrates that can support microbial production of short-chain fatty acids (SCFAs), particularly acetate, propionate, and butyrate. In addition, short-term dietary interventions can rapidly modify microbial diversity and taxonomic composition, demonstrating the responsiveness of the gut microbiota to changes in nutrient supply [4]. Thus, the microbiological effects of individual carbohydrates and lipids need to be considered within the broader macronutrient context in which they are consumed.

Glucose and fructose are the predominant dietary monosaccharides in both human and animal nutrition. Although they provide similar amounts of gross energy (GE), they differ substantially in intestinal absorption, hepatic metabolism, and the amount of carbohydrate potentially reaching the distal intestine [5,6]. Excessive fructose consumption has frequently been associated with impaired intestinal barrier function, altered microbial composition, and metabolic disorders, whereas glucose has been associated with different physiological and microbial responses [7]. Nevertheless, most previous studies have examined the effects of dietary carbohydrates or dietary fat independently, whereas comparatively little attention has been paid to their interaction within the same dietary model.

Dietary fat and carbohydrate composition also influence the relative representation of dominant bacterial phyla, particularly Bacillota and Bacteroidota, as well as overall microbial diversity [8,9]. However, the ecological consequences of progressively replacing dietary lipid with individual monosaccharides under controlled nutritional conditions remain poorly understood. Moreover, most microbiome studies rely primarily on relative abundance data. Because relative abundance is compositional, apparent changes in one taxon may result either from a change in its proportional representation or from reductions in other members of the microbial community [10]. Consequently, relative abundance and sequencing/classified read counts may provide complementary perspectives on treatment-associated differences in microbial community profiles [10,11].

To investigate these questions, we developed a controlled dietary model in which dietary lard was progressively replaced with either glucose or fructose while maintaining a constant protein-to-energy ratio across diets. A carbohydrate-free, lard-rich diet served as the monosaccharide-free control, and all diets contained a minimum of 5% corn oil to ensure an adequate supply of essential fatty acids. This design enabled systematic comparison of both monosaccharide identity and the dietary monosaccharide-to-lard energy ratio.

We hypothesized that the combined presence of dietary lard and monosaccharides would be more strongly associated with cecal microbiota organization than monosaccharide identity alone, whereas glucose and fructose would be associated with differential taxon-specific responses within this dietary framework. The primary objective was to descriptively characterize treatment-level cecal microbiome profiles across controlled dietary gradients in which lard was progressively replaced by glucose or fructose, using sequencing/classified read counts and relative abundance, with additional exploratory assessment of multivariate organization and taxon–taxon co-variation.

## 2. Materials and Methods

### 2.1. Experimental Design and Animals

The present study is based on previously unpublished microbiome data obtained from an animal experiment that formed part of a broader nutritional investigation. The physiological, growth performance, organ weight, and serum metabolic responses of the animals have been reported previously by Szabó et al. [12], whereas the cecal microbiome data presented here have not previously been published.

Seventy-two 10-week-old specific pathogen-free (SPF) male Wistar rats (200–275 g; initial body weight 241.71 ± 17.66 g) were housed individually in wire-bottom cages under controlled environmental conditions (22 ± 2 °C; 12 h light/12 h dark cycle). The animals had free access to the experimental diets and drinking water throughout the study. Following a 3-day acclimatization period, the rats were randomly allocated to nine experimental dietary groups (*n* = 8 per group) with similar mean initial body weights (241.71 ± 17.66 g). The experimental diets were fed for 28 days.

All experimental procedures were approved by the Institutional Animal Care and Use Committee and complied with the applicable national regulations governing the care and use of laboratory animals. The study was conducted in accordance with the ARRIVE guidelines and followed internationally accepted principles for animal experimentation. Clinical characteristics, feed intake, body weight development, organ weights, and serum metabolic variables obtained from the same animals have been reported previously [12] and are therefore not repeated here. The present study is restricted to treatment-associated differences in cecal microbiota composition and ecological organization, together with the humoral immune response measured in the same animals. The present manuscript therefore avoids duplicate presentation of previously published host-level results and focuses on the previously unpublished microbiome dataset.

### 2.2. Experimental Diets

Nine purified experimental diets were formulated to investigate the effects of the progressive replacement of dietary lard with either glucose or fructose. A carbohydrate-free, lard-rich diet (L_6.03_) served as the monosaccharide-free control. In the glucose series, dietary lard was progressively replaced by glucose to generate the GL_5.28_, GL_4.70_, GL_4.23_, and G_3.85_ diets. The corresponding fructose series consisted of the FL_5.28_, FL_4.70_, FL_4.23_, and F_3.85_ diets, in which dietary lard was replaced by fructose according to the same experimental design.

The experimental diets provided carbohydrate-to-fat energy ratios of 5/95, 23/77, 43/57, 64/36, and 86/14. At each energy ratio, the corresponding glucose- and fructose-containing diets differed only in the identity of the monosaccharide, thereby allowing direct comparison of the effects of the two monosaccharides. 

The replacement of dietary lard with glucose or fructose was performed on a gross energy (GE) basis. For each dietary treatment, the gross energy removed by reducing the lard content was replaced by an identical amount of gross energy supplied by either glucose or fructose. Diets were formulated to maintain constant nutrient-to-GE ratios, ensuring that protein, vitamins, minerals, corn oil, and all other essential nutrients were supplied in proportion to the total dietary gross energy. Because lard provides approximately 9 kcal of gross energy per gram, whereas glucose and fructose provide approximately 4 kcal/g, progressively larger amounts of monosaccharide were required to replace the same amount of dietary gross energy supplied by lard. Consequently, the gross energy density of the diets decreased progressively as the proportion of monosaccharide increased.

The diets were therefore intentionally not formulated to be isocaloric on a weight basis. Maintaining identical dietary energy density would have required the incorporation of non-digestible diluents or bulking agents, which could themselves alter the intestinal microbial ecosystem and thereby introduce an additional experimental variable. Instead, the present experimental design maintained constant nutrient-to-gross energy ratios while allowing dietary gross energy density to vary according to the intrinsic energy densities of the replaced nutrients. The percentage composition of the experimental diets shown in Table 1 was calculated from the ingredient quantities obtained after completion of the energy-based formulation procedure. All diets satisfied the nutrient requirements of growing rats.

Accordingly, complete comparison of glucose and fructose effects was performed exclusively between corresponding diets with identical carbohydrate-to-fat energy ratios. Because the experimental diets differed in energy density and macronutrient composition, actual nutrient exposure cannot be inferred from dietary percentages alone. Therefore, mean feed intake data previously reported for the same experimental animals [12] were combined with the calculated dietary formulation to derive treatment-level daily intakes of dietary energy and the principal macronutrient components relevant to the present dietary model. These calculated values are presented in Table 2.

### 2.3. Sample Collection, Microbiome Pooling and Humoral Immune Response

Blood, plasma or serum samples were collected on Days 14 and 28 in glass tubes (non-heparinized or heparinized for serum or plasma, respectively), kept on ice, and then centrifuged (4–8 °C, 10 min, 492 RCF) to obtain the plasma or serum fraction.

The rats were immunized intraperitoneally with ovalbumin (OVA) on Day 0 and received a booster immunization on Day 14 to evaluate primary and secondary humoral immune responses. Serum anti-OVA antibody titers were determined as described previously [13].

Anti-OVA antibody titers were analyzed at the individual-animal level (*n* = 8 per dietary group) using one-way analysis of variance (ANOVA). When the overall ANOVA indicated a significant treatment effect, differences between group means were evaluated using Fisher’s least significant difference (LSD) post hoc test. Differences were considered statistically significant at *p* < 0.05.

At the end of the feeding trial, the animals were euthanized, and cecal contents were collected. To obtain treatment-level microbiome samples, cecal contents from the eight rats within each dietary group were pooled. Briefly, 1 g of cecal content was collected from each animal, yielding 8 g of pooled material per treatment group. The pooled sample was thoroughly homogenized, and a 1 g aliquot was used for DNA extraction.

Samples were maintained at approximately 5 °C during processing, and DNA extraction was initiated within 5 h after sample collection to minimize post-sampling changes in microbial composition.

Pooling was adopted because the objective of the present study was to characterize treatment-level microbiome responses to dietary interventions rather than inter-individual variation. This approach provided a composite microbial community profile for each dietary treatment but did not permit assessment of within-group biological variability. Consequently, the microbiome results were interpreted at the treatment-group level rather than at the level of individual animals, and no inferential statistical comparisons among dietary groups were performed for microbiome outcomes.

### 2.4. DNA Extraction and Shotgun Metagenomic Sequencing

Genomic DNA was extracted from 1 g aliquots of pooled cecal homogenates using the ZR Fecal DNA Kit (Zymo Research, Irvine, CA, USA) according to the manufacturer’s instructions. DNA quantity and quality were assessed before library preparation.

Shotgun metagenomic libraries were prepared using the Ion Xpress™ Plus Fragment Library Kit (Thermo Fisher Scientific, Waltham, MA, USA). DNA fragmentation was performed with the Ion Shear™ Plus Reagents Kit, whereas library amplification, quantification, and emulsion PCR were carried out using Platinum® PCR SuperMix, the Ion Library TaqMan® qPCR Kit, and the Ion PGM™ 200 Xpress Template Kit, respectively. All listed library-preparation reagents were obtained from Thermo Fisher Scientific, Waltham, MA, USA.

Sequencing was performed on an Ion Torrent Personal Genome Machine (PGM™; Thermo Fisher Scientific, Waltham, MA, USA) using Ion 318™ chips (Thermo Fisher Scientific, Waltham, MA, USA) according to the manufacturer’s instructions. Approximately 150,000–520,000 high-quality reads were generated per treatment group, with an average read length of 208 ± 87 bp.

The sequencing depth was used for treatment-level characterization of microbial community composition at the phylum and genus levels and was not intended for detailed species-level analyses.

Raw sequencing data were subsequently subjected to an identical bioinformatic workflow for all treatment groups. Although equal masses of pooled cecal material were processed using the same DNA extraction, library preparation, sequencing, and bioinformatic procedures, the study did not include an internal spike-in standard or an independent measurement of total bacterial load. Classified read counts therefore represent the numbers of sequencing reads assigned to taxa within each library and were not interpreted as estimates of absolute or semi-quantitative bacterial abundance.

Raw sequencing reads were quality-filtered and trimmed using Trimmomatic [14], retaining only reads longer than 50 bp. Host-derived sequences were removed by alignment against the *Rattus norvegicus* reference genome using Bowtie2 with the very-sensitive-local option [15]. This host-decontamination approach was applied to minimize false-positive taxonomic assignments resulting from residual host-derived sequences [16]. Duplicate reads were removed using VSEARCH [17], and the remaining reads were taxonomically classified with Kraken2 (k = 35) [18] against the NCBI reference sequence database [19].

To ensure direct comparability among dietary treatments, identical quality-control procedures, taxonomic classification criteria, reference databases, and bioinformatic workflows were applied to all samples throughout the study.

### 2.5. Taxonomic Data Processing and Descriptive Analysis

Taxonomic analyses focused primarily on the phylum and genus levels because these taxonomic ranks provided the most robust classifications at the sequencing depth achieved in the present study. Dominant phyla were defined as those reaching a relative abundance of at least 1% in at least one dietary treatment.

Both sequencing/classified read count (CR) and relative abundance (RA) profiles were retained for subsequent descriptive analyses. For phylum-level visual comparisons, the CR of each phylum in every dietary treatment was normalized to the total phylum-level CR of the L_6.03_ reference group. The same L_6.03_ total CR was used as the denominator for all phyla and dietary treatments, and 100 was subtracted from the resulting percentage to display deviations in positive and negative directions on a common reference scale:

Normalized CR (%) = [(phylum CR/total phylum-level CR in L_6.03_) × 100] − 100

For genus-level graphical presentation, raw, non-normalized CR values were used. Sequencing-derived CR values were not treated as estimates of bacterial biomass or absolute abundance. Taxonomic profiles, hierarchical clustering, and ecological network characteristics were evaluated to characterize treatment-level differences in cecal microbial community organization.

Because cecal samples were pooled within each dietary treatment before DNA extraction, each dietary treatment was represented by one composite microbiome profile. Consequently, within-group microbiome variance, confidence intervals, and individual-level microbiome inference could not be estimated. This does not preclude treatment-level descriptive analyses, because hierarchical clustering and correlation-network analyses were based on treatment-level profiles and did not incorporate within-treatment variance. These analyses were therefore used to characterize patterns of similarity and co-variation among dietary treatments rather than individual-level associations.

### 2.6. Microbiome Data and Statistical Analysis

Microbiome data were analyzed descriptively at the treatment level because each dietary group was represented by one pooled cecal microbiome profile. Humoral immune responses were analyzed at the individual-animal level (*n* = 8 per group) using one-way ANOVA followed, when significant, by Fisher’s LSD test (*p* < 0.05). No additional inferential multivariate analysis, including principal component analysis intended for group-level inference, was performed because the microbiome dataset contained only one pooled profile per dietary treatment.

Exploratory taxon–taxon correlation networks were constructed separately for the glucose and fructose dietary series using Konf00-normalized treatment-level taxonomic data across the five dietary treatments within each series (*n* = 5; df = 3). Pearson correlations were calculated separately at the phylum and genus levels. For all four taxon–taxon networks, correlations meeting the nominal two-tailed *p* < 0.05 criterion (|*r*| ≥ 0.878) were retained. Taxa were additionally filtered according to their maximum relative abundance within the corresponding dietary series, using a threshold of ≥0.2% for the glucose phylum and genus networks and the fructose phylum network, and ≥1.0% for the fructose genus network. Only taxa participating in at least one retained correlation after abundance filtering were displayed. No adjustment for multiple comparisons was applied. The resulting networks were interpreted as exploratory patterns of treatment-level co-variation rather than as confirmatory associations or evidence of direct ecological or causal interactions.

Exploratory correlation networks were visualized using yEd Graph Editor (version 3.25.1) [20]. Positive and negative taxon–taxon correlations were represented by black and red edges, respectively. Numerical edge labels indicate Pearson’s *r*. Network-centrality values were used descriptively to characterize the relative structural positions of taxa within each displayed network and were not interpreted as evidence of causal ecological importance.

For cross-representation analysis, Pearson correlations were calculated between phylum-level classified-read (CR) and relative-abundance (RA) treatment-response profiles separately within the glucose and fructose dietary series. The original, non-normalized phylum-level CR values were used for this analysis. Correlations were calculated across the five treatment-level profiles within each series (*n* = 5; df = 3), and CR–RA associations meeting the nominal two-tailed *p* < 0.05 criterion (|*r*| ≥ 0.878) were retained. Only CR–RA associations were included; CR–CR and RA–RA correlations were excluded. No adjustment for multiple comparisons was applied. The resulting networks were interpreted as descriptive, hypothesis-generating representations of cross-representation treatment-level co-variation. Positive and negative correlations were represented by blue and red edges, respectively.

Hierarchical clustering of dominant bacterial phyla was performed independently using sequencing/classified read-count and relative-abundance data in R with the pheatmap package [21]. Values were standardized as z-scores before clustering. For both data representations, two-way hierarchical clustering was performed using Euclidean distance and complete-linkage clustering, with both bacterial phyla (rows) and dietary treatments (columns) hierarchically clustered.

### 2.7. Use of Artificial Intelligence

During the preparation and revision of this manuscript, the authors used ChatGPT (GPT-5, OpenAI) to assist with language editing, improving clarity and readability, refining the presentation of scientific arguments, and improving the organization of the manuscript. ChatGPT was not used for study design, data generation, or statistical analysis. All AI-assisted content was critically reviewed and verified by the authors.

## 3. Results and Discussion

### 3.1. Interpretation of Relative Abundance and Sequencing/Classified Read Counts

Microbiome data can be evaluated using sequencing/classified read counts (CRs) and relative abundance (RA) which may provide distinct but complementary information.

Absolute quantification methods, such as quantitative metagenomic profiling with appropriate calibration, can estimate total microbial abundance [22,23,24]. Such quantification was not performed in the present study. Although equal masses of pooled cecal material were processed using the same laboratory and bioinformatic workflow, variation in DNA extraction, library preparation, sequencing efficiency, host-to-microbial DNA ratios, and other technical factors cannot be excluded. CRs therefore represent sequencing-derived observations rather than absolute or semi-quantitative bacterial abundance.

Relative abundance is widely used to describe microbial community composition; however, because it represents proportional data, its interpretation may be misleading when substantial differences exist in the total number of detectable microbial sequences among treatment groups [10,25]. Under such conditions, an apparent increase in the relative abundance of a taxon may simply reflect a reduction in other community members rather than a true increase in its read representation [10,25,26]. Accordingly, CRs and RA were retained as two descriptive representations of the observed sequencing profiles. CRs preserve the number of reads assigned to individual taxa within each library, whereas RA describes the proportional distribution of classified reads within a taxonomic level. Neither measure provides absolute bacterial abundance in the absence of quantitative calibration [10,22,23,24].

### 3.2. Total Sequencing/Classified Read Counts

Relative to the L_6.03_ reference profile, all mixed lard–monosaccharide profiles showed lower phylum-level CRs (Table 3). The reductions were 72.0%, 82.2%, and 53.7% in GL_5.28_, GL_4.70_, and GL_4.23_, respectively, and 76.1%, 78.5%, and 83.1% in FL_5.28_, FL_4.70_, and FL_4.23_. The lowest CR values occurred in the mixed dietary profiles rather than at either endpoint. Compared with L_6.03_, phylum-level CRs were 25.4% higher in G_3.85_ and 15.5% lower in F_3.85_.

**Figure 1:**
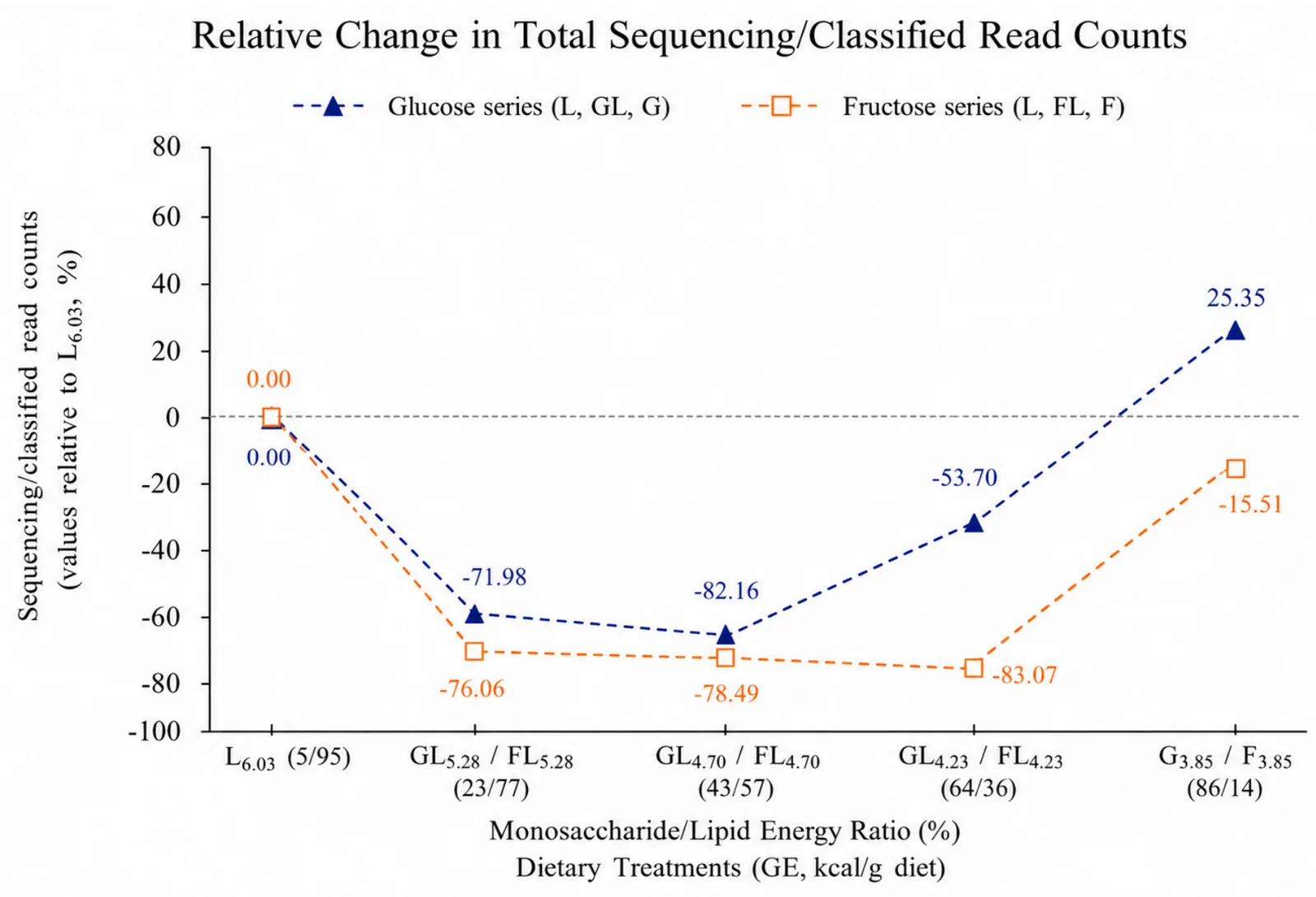
Relative change in total sequencing/classified read counts across the glucose- and fructose-containing dietary series, expressed relative to the pooled L_6.03_ reference sample. Each point represents one pooled cecal microbiome sample; connecting lines are for visual guidance only. Abbreviations: L, lard; GL, glucose + lard; G, glucose; FL, fructose + lard; F, fructose; GE, gross energy.

Progressive replacement of dietary lard with either glucose or fructose was associated with a pronounced non-linear pattern in total CRs (Figure 1). Higher CRs in L_6.03_ and the lard-free endpoints, together with lower values in several mixed diets, defined this pattern. Its reproducibility requires confirmation using independently sequenced biological replicates. Because all samples were collected, processed, sequenced, and analyzed using identical procedures, the observed differences are described as treatment-associated differences in classified microbial read profiles.

### 3.3. Phylum Bacillota

Bacillota (formerly Firmicutes) is a dominant bacterial phylum of the rat cecal microbiota and, together with Bacteroidota, constitutes a major proportion of the bacterial community. Members of this phylum play important roles in the fermentation of complex dietary substrates and include major producers of short-chain fatty acids (SCFAs), particularly butyrate, which contributes to intestinal barrier integrity, immune homeostasis, and host energy metabolism [27,28]. Dietary macronutrient composition can modify gut microbial composition and diversity [29]. Dietary fat quantity and quality have been associated with changes in microbial community structure [30], whereas alterations in dietary carbohydrate and fat content have also been associated with changes in the relative abundance of major bacterial taxa, including Bacillota and Bacteroidota [9]. The magnitude and direction of these responses vary with dietary composition, experimental model, and study conditions [9,29,30].

**Figure 2:**
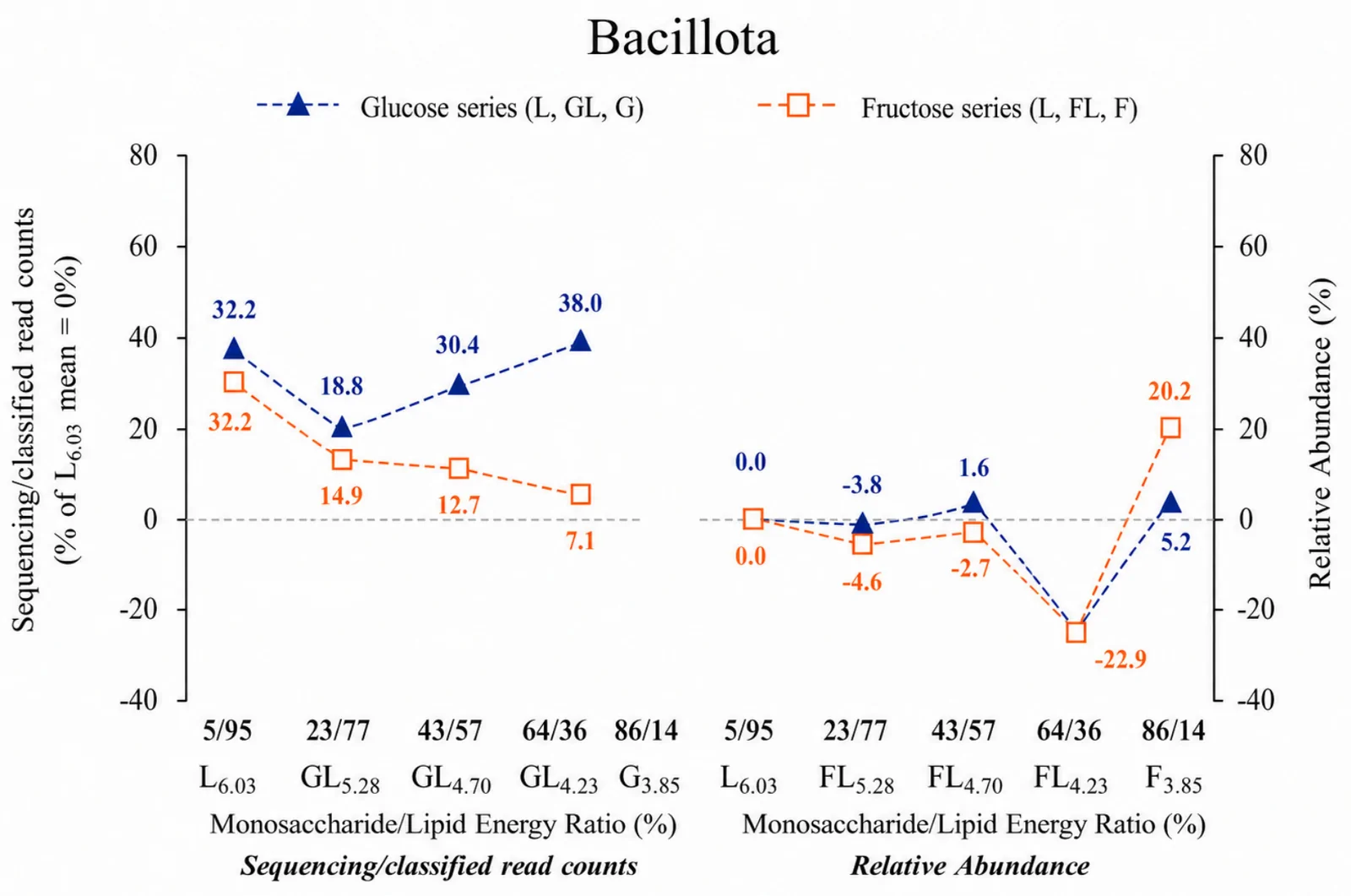
Treatment-level sequencing/classified read counts and relative abundance of Bacillota across the glucose- and fructose-containing dietary series. Read-count values are expressed as percentage changes relative to the mean value of the L_6.03_ reference group (L_6.03_ = 0%). Each point represents one pooled cecal microbiome sample; connecting lines are for visual guidance only. Abbreviations: L, lard; GL, glucose + lard; G, glucose; FL, fructose + lard; F, fructose.

Classified read counts: In contrast to RA, CRs showed a biphasic pattern (Figure 2). Compared with the control group (15,512 classified reads), CRs declined markedly in all mixed dietary groups, reaching a minimum at the 43/57 ratio in the glucose series and at the 64/36 ratio in the fructose series. CRs subsequently recovered as dietary lard was progressively replaced and exceeded the control value in both lard-free diets, reaching 18,297 and 20,790 classified reads in G_3.85_ and F_3.85_, respectively.

Relative abundance: Progressive replacement of dietary lard with glucose or fructose was associated with marked differences in Bacillota RA (Figure 2). In the carbohydrate-free control group (L_6.03_), RA was 32.2%. In all mixed lard–monosaccharide diets, it remained above the control value, ranging from 62.9% to 67.6% in the glucose series and from 42.5% to 63.0% in the fructose series. Under lard-free conditions, however, the two monosaccharides diverged: RA was 30.2% in G_3.85_ but 50.4% in F_3.85_, indicating that differences between glucose and fructose became more apparent when monosaccharides constituted the predominant dietary energy source.

Integrated interpretation: RA and CRs showed partly divergent patterns for Bacillota [22,23,24,25,26]. In the mixed lard–monosaccharide diets, higher RA was accompanied by lower CRs, indicating that greater proportional representation did not necessarily reflect an increase in classified-read representation of this phylum. Under lard-free conditions, CRs exceeded the control value in both dietary series, whereas RA increased in F_3.85_ but remained close to the control level in G_3.85_. Together, these patterns suggest that the Bacillota response was associated with the changing lard–monosaccharide context rather than with progressive monosaccharide replacement alone, while the increasing divergence between glucose and fructose as dietary lard was reduced suggests an additional association with monosaccharide identity.

### 3.4. Phylum Bacteroidota

Bacteroidota (formerly Bacteroidetes) is a dominant bacterial phylum of the rat cecal microbiota and plays an important role in the degradation of complex carbohydrates reaching the large intestine [31,32]. Members of this phylum contribute substantially to the production of short-chain fatty acids (SCFAs), particularly acetate and propionate, thereby supporting intestinal homeostasis and host metabolism [27,28,33,34]. Dietary macronutrient composition can modify gut microbial composition and diversity [29]. Dietary fat quantity and quality have been associated with differences in microbial community structure [30], whereas alterations in dietary carbohydrate and fat content have also been associated with changes in the relative abundance of major bacterial taxa, including Bacillota and Bacteroidota [9]. The magnitude and direction of these responses vary with dietary composition, experimental model, and study conditions [9,29,30].

**Figure 3:**
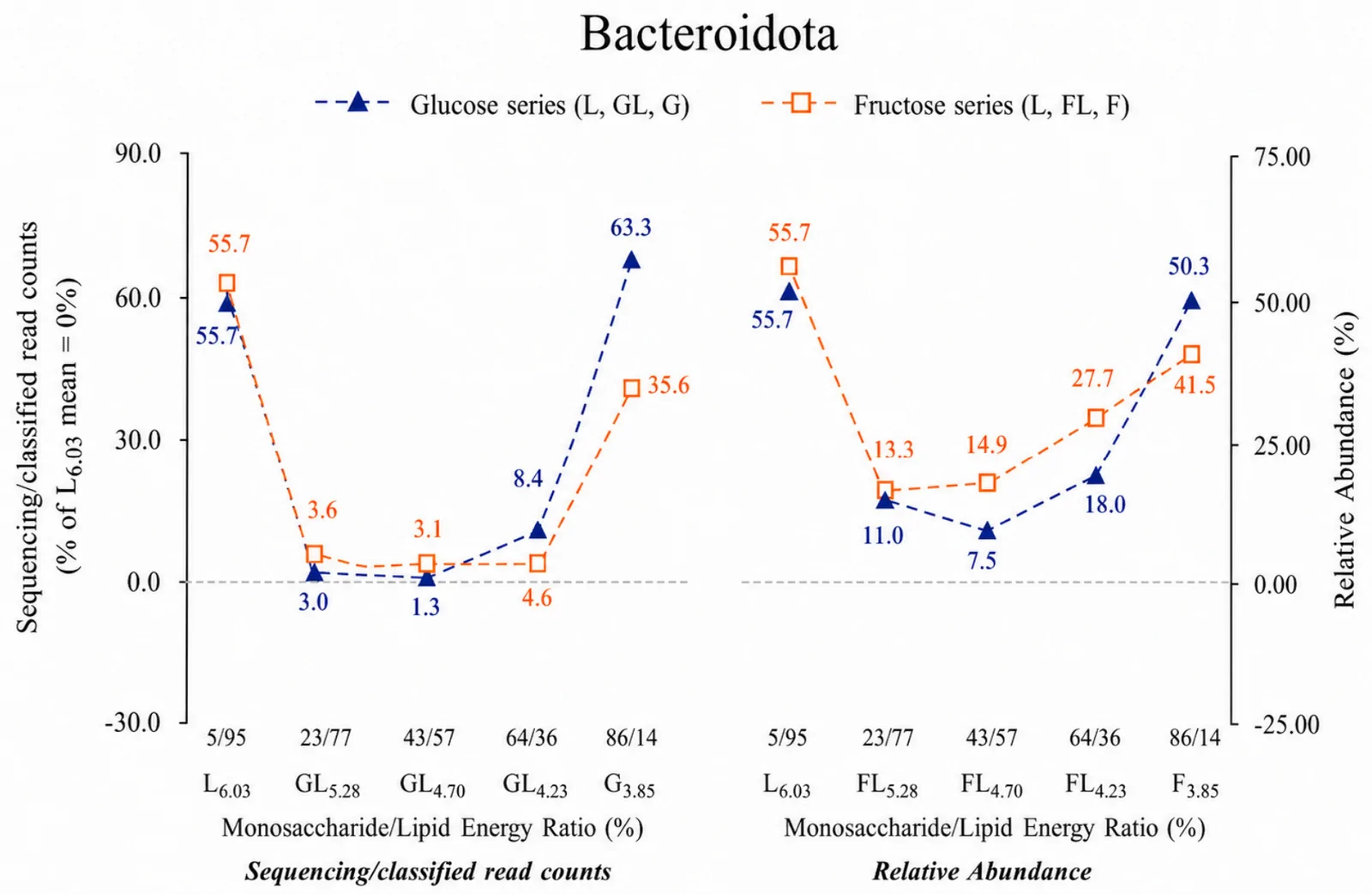
Treatment-level sequencing/classified read counts and relative abundance of Bacteroidota across the glucose- and fructose-containing dietary series. Read-count values are expressed relative to the L_6.03_ reference value (L_6.03_ = 0%). Each point represents one pooled cecal microbiome sample; connecting lines are for visual guidance only. Abbreviations: L, lard; GL, glucose + lard; G, glucose; FL, fructose + lard; F, fructose.

Classified read counts: CRs closely paralleled RA (Figure 3). Compared with the carbohydrate-free control (26,823 classified reads), CRs declined markedly in all mixed dietary groups, reaching the lowest values at intermediate dietary compositions. CRs increased again when dietary lard was completely replaced by monosaccharides, reaching 30,499 reads in G_3.85_ (13.7% above the control) and 17,124 reads in F_3.85_ (36.2% below the control). Thus, RA and CRs showed similar non-linear patterns, with the lowest values in the mixed lard–monosaccharide diets.

Relative abundance: Bacteroidota RA differed markedly across dietary treatments (Figure 3). In the carbohydrate-free control group (L_6.03_), RA was 55.7%. It declined sharply in all mixed lard–monosaccharide diets, reaching 7.5–18.0% in the glucose series and 14.9–27.7% in the fructose series. Following complete replacement of dietary lard by monosaccharides, it recovered to 50.3% in G_3.85_ and 41.5% in F_3.85_. Thus, the lowest RA occurred in the mixed lard–monosaccharide diets.

Integrated interpretation: In contrast to Bacillota, Bacteroidota showed concordant patterns in RA and CRs, indicating that lower proportional representation was accompanied by fewer detectable classified reads. Both measures were lowest in the mixed lard–monosaccharide diets and recovered at the lard-free endpoints, particularly in the glucose series. This concordant non-linear pattern further supports an association with the lard–monosaccharide dietary context, while the divergence between G_3.85_ and F_3.85_ suggests that differences between glucose and fructose became more apparent under lard-free conditions.

### 3.5. Bacillota-to-Bacteroidota Ratio

Because the Bacillota-to-Bacteroidota (B/B) ratio is mathematically identical when calculated from CRs or RA within the same sample, it is invariant to normalization and reflects the relative balance between these two dominant phyla. This ratio has been used to characterize diet-associated shifts in gut microbial composition. Increased B/B ratios have been reported in some experimental and clinical settings associated with obesity and diabetes; however, their direction and magnitude vary substantially among hosts, experimental models, and dietary interventions [35,36,37,38]. The B/B ratio should therefore be interpreted as a context-dependent ecological indicator rather than a universal biomarker of dysbiosis.

**Figure 4:**
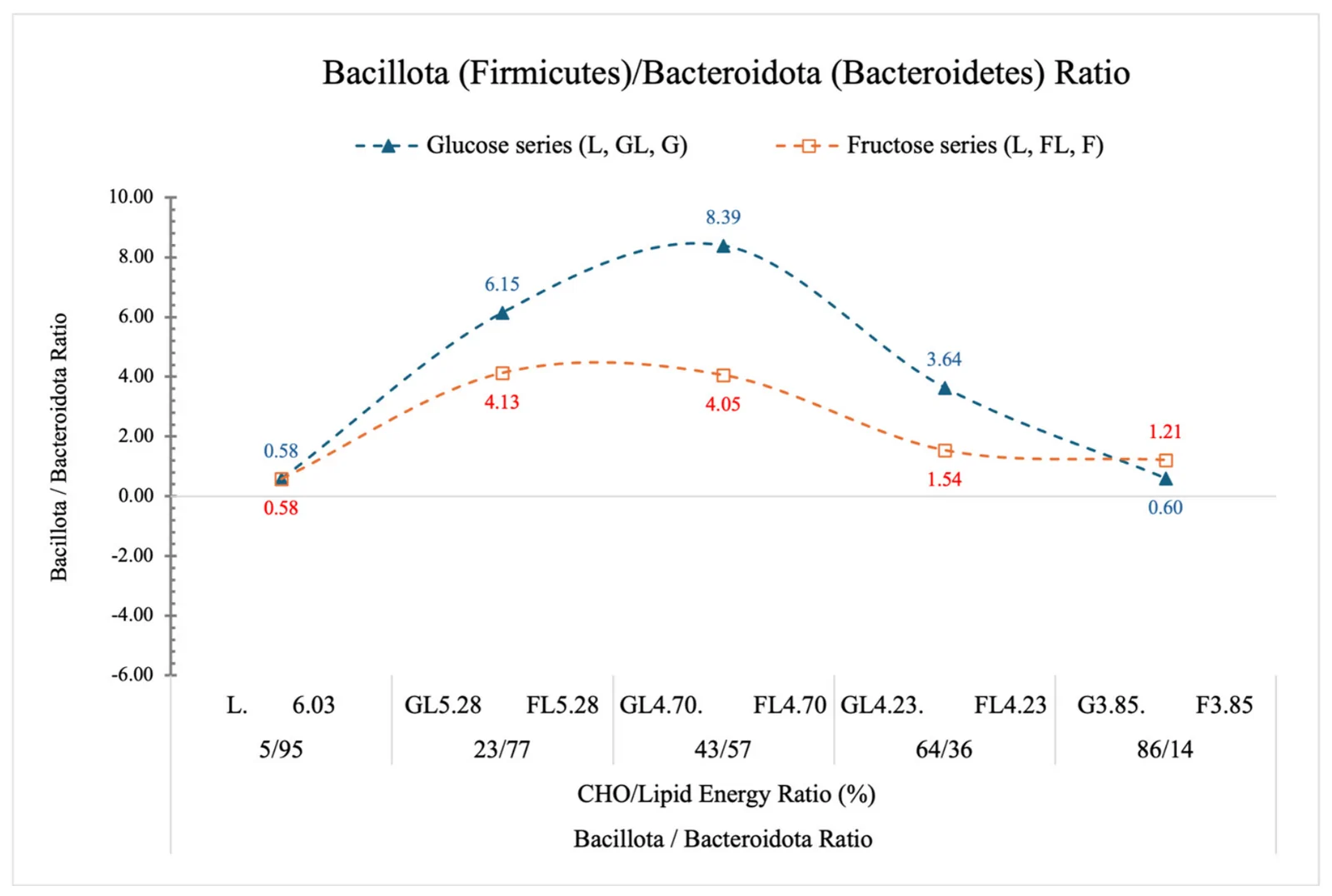
Treatment-level Bacillota-to-Bacteroidota (B/B) ratio across the glucose- and fructose-containing dietary series. Each point represents one pooled cecal microbiome sample; connecting lines are for visual guidance only. Abbreviations: B/B, Bacillota-to-Bacteroidota ratio; L, lard; GL, glucose + lard; G, glucose; FL, fructose + lard; F, fructose.

The carbohydrate-free, lard-rich control diet (L_6.03_) exhibited a low B/B ratio (0.58), reflecting the predominance of Bacteroidota over Bacillota (Figure 4). The ratio increased markedly in the mixed lard–monosaccharide diets, particularly in the glucose series, reaching a maximum of 8.39 in GL_4.70_. The corresponding fructose-containing diets showed lower ratios, ranging from 1.54 to 4.13. These elevated B/B ratios coincided with marked reductions in CRs, linking the shift in the balance between the two dominant phyla to broader differences in the microbial read profiles. 

Complete replacement of dietary lard produced a different pattern. The B/B ratio decreased to 0.60 in G_3.85_ and 1.21 in F_3.85_, approaching the control value, while CRs recovered at both lard-free endpoints. Thus, the B/B ratio showed a pronounced non-linear association with dietary composition, with the highest values occurring in the mixed lard–monosaccharide diets rather than at either endpoint.

Integrated interpretation: The B/B ratio summarizes the opposing Bacillota and Bacteroidota patterns and, because it is invariant to normalization, is independent of whether it is calculated from RA or CRs. Its marked increase in the mixed diets, followed by a decline toward the control value at the lard-free endpoints, further suggests that the balance between these dominant phyla was associated with the lard–monosaccharide dietary context rather than with progressive monosaccharide replacement alone. The lower endpoint ratio in G_3.85_ than in F_3.85_ is also consistent with the increasing differentiation between the glucose and fructose series as dietary lard was reduced.

### 3.6. Phylum Pseudomonadota

**Figure 5:**
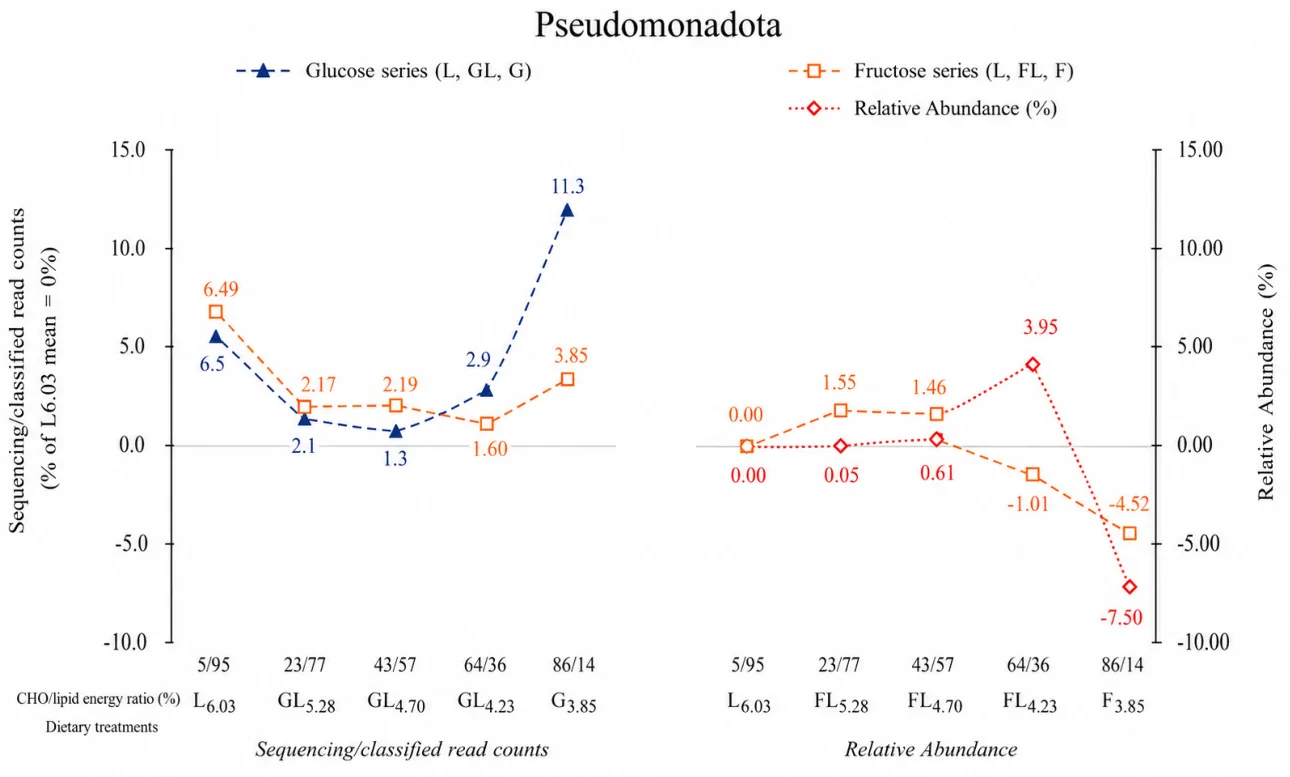
Treatment-level sequencing/classified read counts and relative abundance of Pseudomonadota across the glucose- and fructose-containing dietary series. Read-count values are expressed relative to the mean value of the L_6.03_ reference group (L_6.03_ = 0%). Each point represents one pooled cecal microbiome sample; connecting lines are for visual guidance only. Abbreviations: L, lard; GL, glucose + lard; G, glucose; FL, fructose + lard; F, fructose.

**Figure 6:**
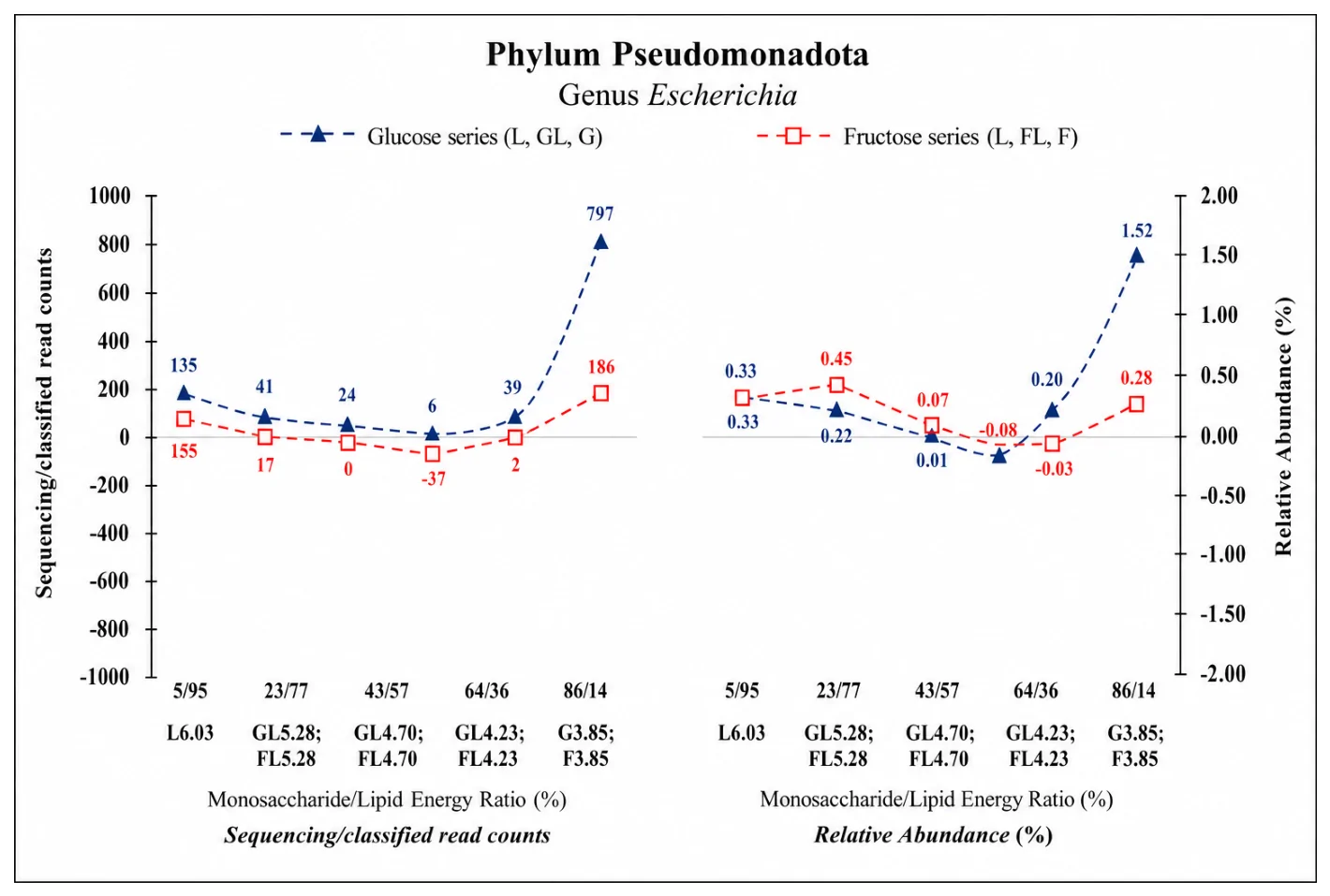
Treatment-level sequencing/classified read counts and relative abundance of *Escherichia* (phylum Pseudomonadota) across the glucose- and fructose-containing dietary series. Each point represents one pooled cecal microbiome sample; connecting lines are for visual guidance only. Abbreviations: L, lard; GL, glucose + lard; G, glucose; FL, fructose + lard; F, fructose.

Pseudomonadota (formerly Proteobacteria) generally constitutes a minor component of the healthy gut microbiota, but increased representation has been associated with microbial dysbiosis and intestinal inflammation. Many members possess lipopolysaccharide (LPS)-containing outer membranes, and the phylum includes opportunistic pathogens that may expand under dysbiotic conditions [39,40,41]. CRs and RA for Pseudomonadota and *Escherichia* are presented in Figure 5 and Figure 6, respectively.

Relative abundance: Pseudomonadota RA showed a non-linear pattern across both dietary series (Figure 5). In the glucose series, RA increased from 6.49% in L_6.03_ to 7.61% and 8.69% in GL_5.28_ and GL_4.70_, declined to 5.67% in GL_4.23_, and increased again to 9.01% in G_3.85_. In the fructose series, RA increased to 9.16–10.16% in the mixed diets before declining to 4.49% in F_3.85_. Thus, the two dietary series showed distinct patterns, with the greatest divergence between glucose and fructose at the lard-free endpoints.

Classified read counts: CRs showed a different pattern from RA (Figure 5). In both dietary series, CRs declined in the mixed lard–monosaccharide diets relative to L_6.03_, with the lowest values observed at intermediate dietary compositions. At the lard-free endpoints, CRs increased markedly in G_3.85_ but showed only partial recovery in F_3.85_. Thus, the CR pattern differed between the two dietary series, with the greatest divergence occurring under lard-free conditions.

At the genus level, *Escherichia* showed a pronounced divergence between the glucose and fructose series, particularly under lard-free conditions (Figure 6). CRs decreased from 135 in L_6.03_ to low values in the mixed diets of both series. At the lard-free endpoints, however, CRs increased markedly to 797 in G_3.85_ compared with 106 in F_3.85_. This endpoint divergence suggests that the *Escherichia* pattern became increasingly associated with monosaccharide identity as dietary lard was reduced.

Integrated interpretation: Pseudomonadota showed partly divergent patterns in RA and CRs. In the mixed lard–monosaccharide diets, RA was generally maintained or increased despite lower CRs, indicating that greater proportional representation did not necessarily reflect an increase in detectable classified reads. The glucose and fructose series also differed, particularly under lard-free conditions, where both RA and CRs were higher in G_3.85_ than in F_3.85_. Thus, the relationship between RA and CRs varied with dietary composition, while the increasing glucose–fructose divergence as dietary lard was reduced suggests an additional association with monosaccharide identity.

At the genus level, *Escherichia* reinforced this glucose–fructose divergence. Both CRs and RA increased markedly in G_3.85_ but remained substantially lower in F_3.85_, showing that the endpoint difference was evident with both sequencing-derived representations. Increased representation of Pseudomonadota, particularly members of Enterobacteriaceae, has been associated with microbial dysbiosis and gastrointestinal inflammation [39,40]. However, the taxonomic pattern alone does not establish functional or pathological consequences.

Collectively, these findings indicate that Pseudomonadota and *Escherichia* responses were associated with both the lard–monosaccharide dietary context and monosaccharide identity, with the clearest glucose–fructose differentiation occurring under lard-free conditions [42].

### 3.7. Phylum Actinomycetota

**Figure 7:**
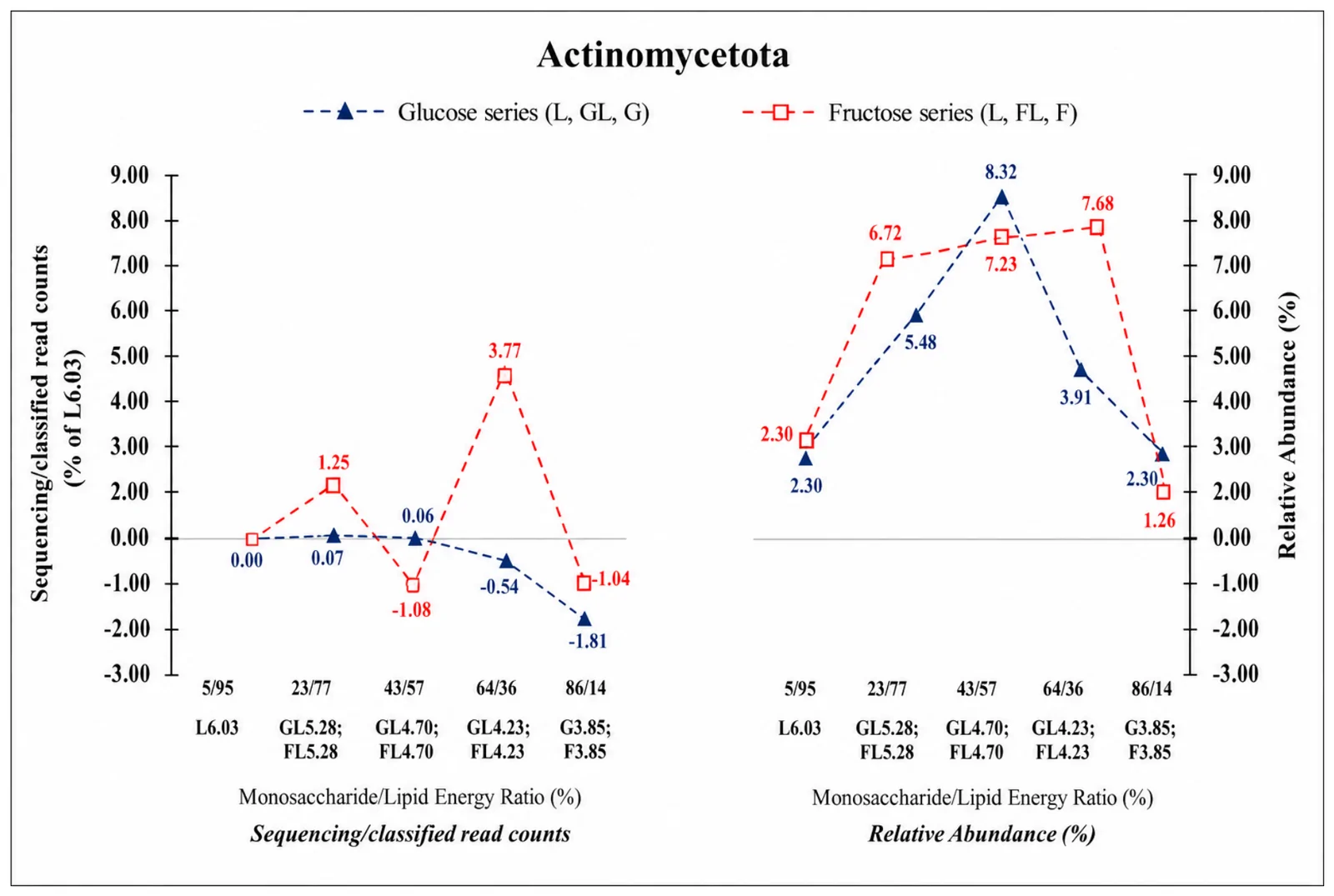
Treatment-level sequencing/classified read counts and relative abundance of Actinomycetota across the glucose- and fructose-containing dietary series. Read-count values are expressed relative to the mean value of the L_6.03_ reference group (L_6.03_ = 0%). Each point represents one pooled cecal microbiome sample; connecting lines are for visual guidance only. Abbreviations: L, lard; GL, glucose + lard; G, glucose; FL, fructose + lard; F, fructose.

Actinomycetota are Gram-positive bacteria involved in gut homeostasis, fermentation, and microbial community stability [43]. Dietary composition can influence their abundance and that of beneficial genera such as *Bifidobacterium* [44,45,46,47]. In the present study, Actinomycetota showed smaller phylum-level changes than Bacillota, Bacteroidota, and Pseudomonadota. CRs and RA are presented in Figure 7, followed by genus-level responses of *Adlercreutzia*, *Streptomyces*, and *Bifidobacterium*.

Classified read counts: CRs showed moderate differences across the dietary series (Figure 7). In the glucose series, CRs decreased in the mixed lard–monosaccharide diets before increasing above the control level in G_3.85_. In the fructose series, CRs remained below the control throughout and declined further toward F_3.85_. Thus, the two series showed increasingly different CR patterns toward the lard-free endpoints.

Relative abundance: Actinomycetota RA showed a more pronounced non-linear pattern. From 2.30% in L_6.03_, RA increased in the mixed diets, reaching 8.32% in GL_4.70_ and 7.68% in FL_4.23_. At the lard-free endpoints, it returned to 2.30% in G_3.85_ and declined to 1.26% in F_3.85_. Thus, the highest proportional representation occurred in the mixed lard–monosaccharide diets rather than at the endpoints.

Integrated interpretation: CRs and RA showed partly divergent patterns. The increased RA in several mixed diets was not accompanied by higher CRs, indicating that greater proportional representation did not necessarily reflect an increase in detectable classified reads. At the lard-free endpoints, both measures distinguished the two dietary series: CRs recovered in G_3.85_ but continued to decline toward F_3.85_, while RA returned to the control level in G_3.85_ and fell below it in F_3.85_. Together, these findings suggest that the Actinomycetota response was associated with the changing lard–monosaccharide dietary context, with differences between glucose and fructose becoming most apparent under lard-free conditions, where both CRs and RA were lower in F_3.85_ than in G_3.85_. Given the established sensitivity of Actinomycetota to dietary substrate availability [44,45,46,47], the non-linear response further suggests that their representation was related to the overall macronutrient environment rather than to progressive monosaccharide replacement alone.

**Figure 8:**
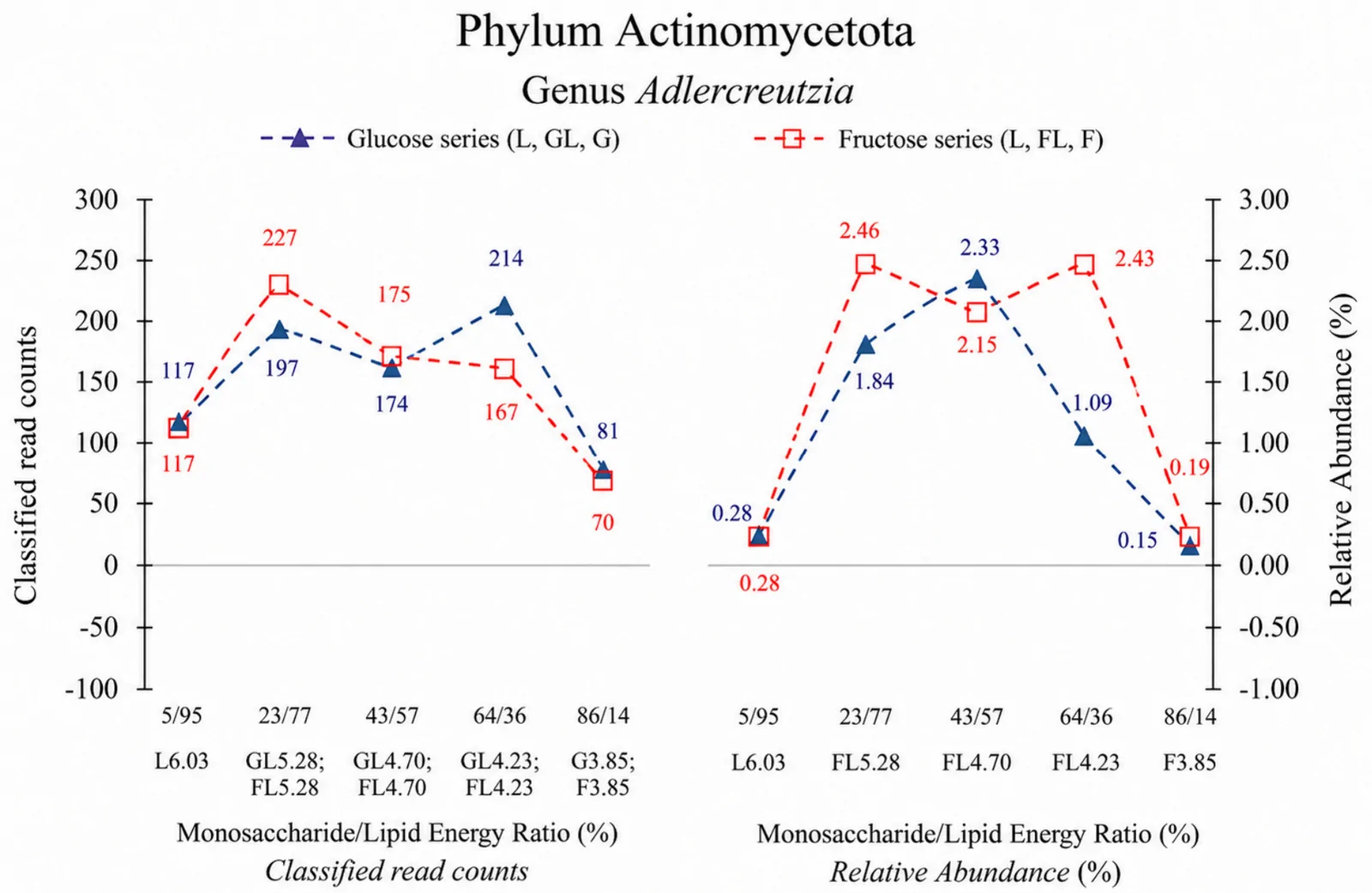
Treatment-level sequencing/classified read counts and relative abundance of *Adlercreutzia* (phylum Actinomycetota) across the glucose- and fructose-containing dietary series. Genus-level CR values represent raw, non-normalized classified read counts. Each point represents one pooled cecal microbiome sample; connecting lines are for visual guidance only. Abbreviations: L, lard; GL, glucose + lard; G, glucose; FL, fructose + lard; F, fructose.

At the genus level, the Actinomycetota response was heterogeneous, indicating that phylum-level patterns did not uniformly reflect the responses of individual taxa. Therefore, *Adlercreutzia*, *Streptomyces*, and *Bifidobacterium* were examined separately to characterize genus-specific patterns across the dietary series. *Adlercreutzia* showed a non-linear response across both dietary series (Figure 8).

Classified read counts: CRs increased from 117 reads in L_6.03_ to higher values in all mixed lard–monosaccharide diets, ranging from 174 to 214 reads in the glucose series and from 167 to 227 reads in the fructose series. At the lard-free endpoints, CRs declined below the control value, reaching 81 reads in G_3.85_ and 70 reads in F_3.85_.

Relative abundance: RA showed a broadly similar pattern. From 0.28% in L_6.03_, it increased in all mixed diets, reaching 1.84–2.33% in the glucose series and 2.15–2.46% in the fructose series. At the lard-free endpoints, RA declined to 0.15% in G_3.85_ and 0.19% in F_3.85_.

Integrated interpretation: CRs and RA showed broadly concordant patterns for *Adlercreutzia*, with higher values in the mixed lard–monosaccharide diets followed by a decline below the control level at both lard-free endpoints. This genus-level response differed from the broader Actinomycetota pattern, in which increased RA in the mixed diets was not generally accompanied by higher CRs. Differences between matched glucose and fructose diets varied in magnitude and direction across the dietary gradient, with no consistent separation between the two series. Together, these findings suggest that the *Adlercreutzia* response was associated more closely with the changing lard–monosaccharide dietary context than with monosaccharide identity alone.

**Figure 9:**
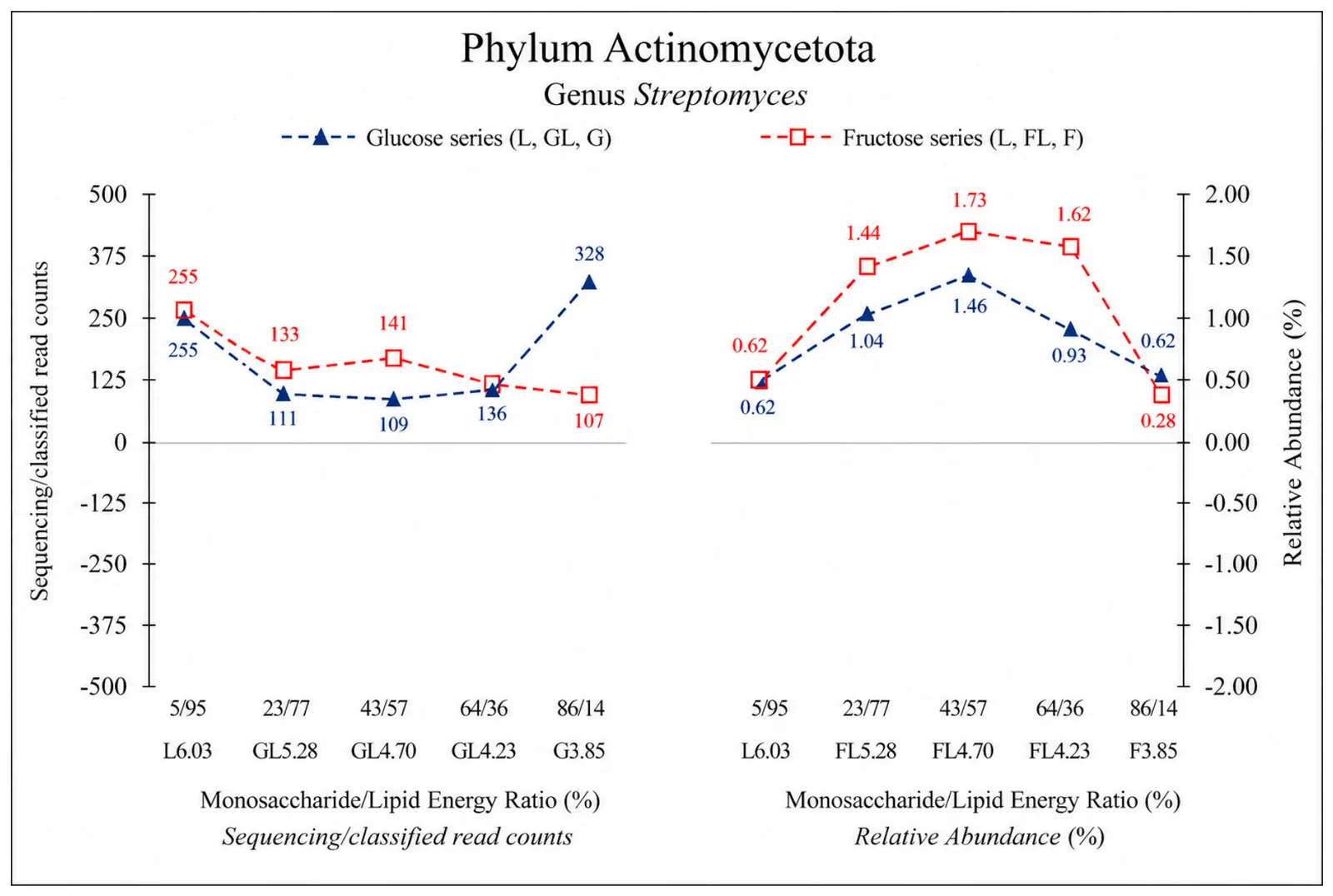
Treatment-level sequencing/classified read counts and relative abundance of *Streptomyces* (phylum Actinomycetota) across the glucose- and fructose-containing dietary series. Genus-level CR values represent raw, non-normalized classified read counts. Each point represents one pooled cecal microbiome sample; connecting lines are for visual guidance only. Abbreviations: L, lard; GL, glucose + lard; G, glucose; FL, fructose + lard; F, fructose.

In contrast to the broadly concordant CR and RA patterns observed for *Adlercreutzia*, *Streptomyces* showed a distinct relationship between the two measures across the dietary series (Figure 9).

Classified read counts: From 255 reads in L_6.03_, CRs decreased in all mixed lard–monosaccharide diets, reaching 109–136 reads in the glucose series and 133–141 reads in the fructose series. At the lard-free endpoints, however, the two series diverged: CRs increased to 328 reads in G_3.85_ but remained below the control level at 107 reads in F_3.85_.

Relative abundance: In contrast to CRs, RA increased in the mixed diets. From 0.62% in L_6.03_, it reached 1.04–1.46% in the glucose series and 1.44–1.73% in the fructose series. At the lard-free endpoints, RA returned to the control value (0.62%) in G_3.85_ and declined to 0.28% in F_3.85_.

Integrated interpretation: CRs and RA therefore showed divergent patterns for *Streptomyces* in the mixed lard–monosaccharide diets, where higher RA occurred despite lower CRs. This contrasts with *Adlercreutzia*, for which higher values in the mixed diets were generally evident with both measures, and illustrates that genera within the same phylum did not respond uniformly to dietary composition. The glucose and fructose series also diverged toward the lard-free endpoints: in G_3.85_, CRs exceeded the control while RA returned to the control level, whereas both measures were lower in F_3.85_. Together, these patterns suggest that the *Streptomyces* response was associated with the changing lard–monosaccharide context, with differences between glucose and fructose becoming most apparent under lard-free conditions.

**Figure 10:**
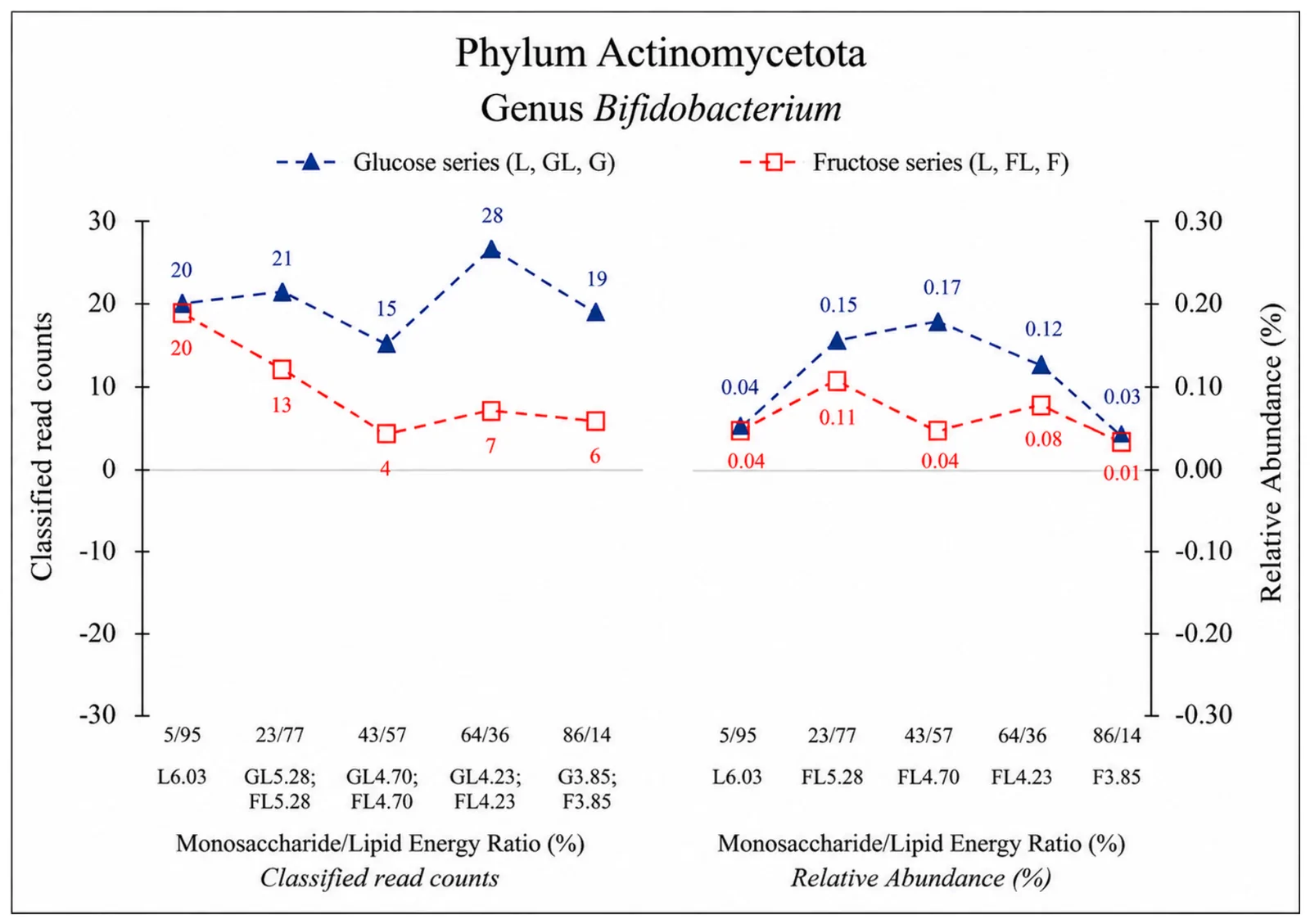
Treatment-level sequencing/classified read counts and relative abundance of *Bifidobacterium* (phylum Actinomycetota) across the glucose- and fructose-containing dietary series. Genus-level CR values represent raw, non-normalized classified read counts. Each point represents one pooled cecal microbiome sample; connecting lines are for visual guidance only. Abbreviations: L, lard; GL, glucose + lard; G, glucose; FL, fructose + lard; F, fructose.

*Bifidobacterium*, although present at low relative abundance, showed a more consistent separation between the glucose and fructose series than *Adlercreutzia* or *Streptomyces* (Figure 10).

Classified read counts: CRs were consistently higher in the glucose-containing diets than in the corresponding fructose-containing diets. From 20 reads in L_6.03_, CRs remained between 15 and 28 reads across the mixed glucose-containing diets, reaching the highest value in GL_4.23_, whereas the corresponding fructose-containing diets contained only 4–13 reads. At the lard-free endpoints, CRs were 19 reads in G_3.85_ and 6 reads in F_3.85_.

Relative abundance: RA showed a broadly concordant glucose–fructose separation. From 0.04% in L_6.03_, RA increased to 0.12–0.17% in the mixed glucose-containing diets, compared with 0.04–0.11% in the corresponding fructose-containing diets. At the lard-free endpoints, RA declined to 0.03% in G_3.85_ and 0.01% in F_3.85_.

Integrated interpretation: In contrast to the more variable genus-level patterns of *Adlercreutzia* and *Streptomyces, Bifidobacterium* showed a relatively consistent separation between the glucose and fructose series, with both CRs and RA generally higher in the glucose-containing diets. The concordance between the two measures indicates that the glucose–fructose difference was evident in both proportional representation and detectable classified reads. Although *Bifidobacterium* represented less than 1% of the microbial community, its distinct pattern illustrates that genera with low relative abundance may show dietary associations that are not apparent from phylum-level *Actinomycetota* responses [44,45,46,47]. Thus, genus-level analysis revealed an additional layer of taxon-specific variation, with *Bifidobacterium* showing a more consistent association with monosaccharide identity than *Adlercreutzia* or *Streptomyces*.

### 3.8. Phylum Thermodesulfobacteriota

**Figure 11:**
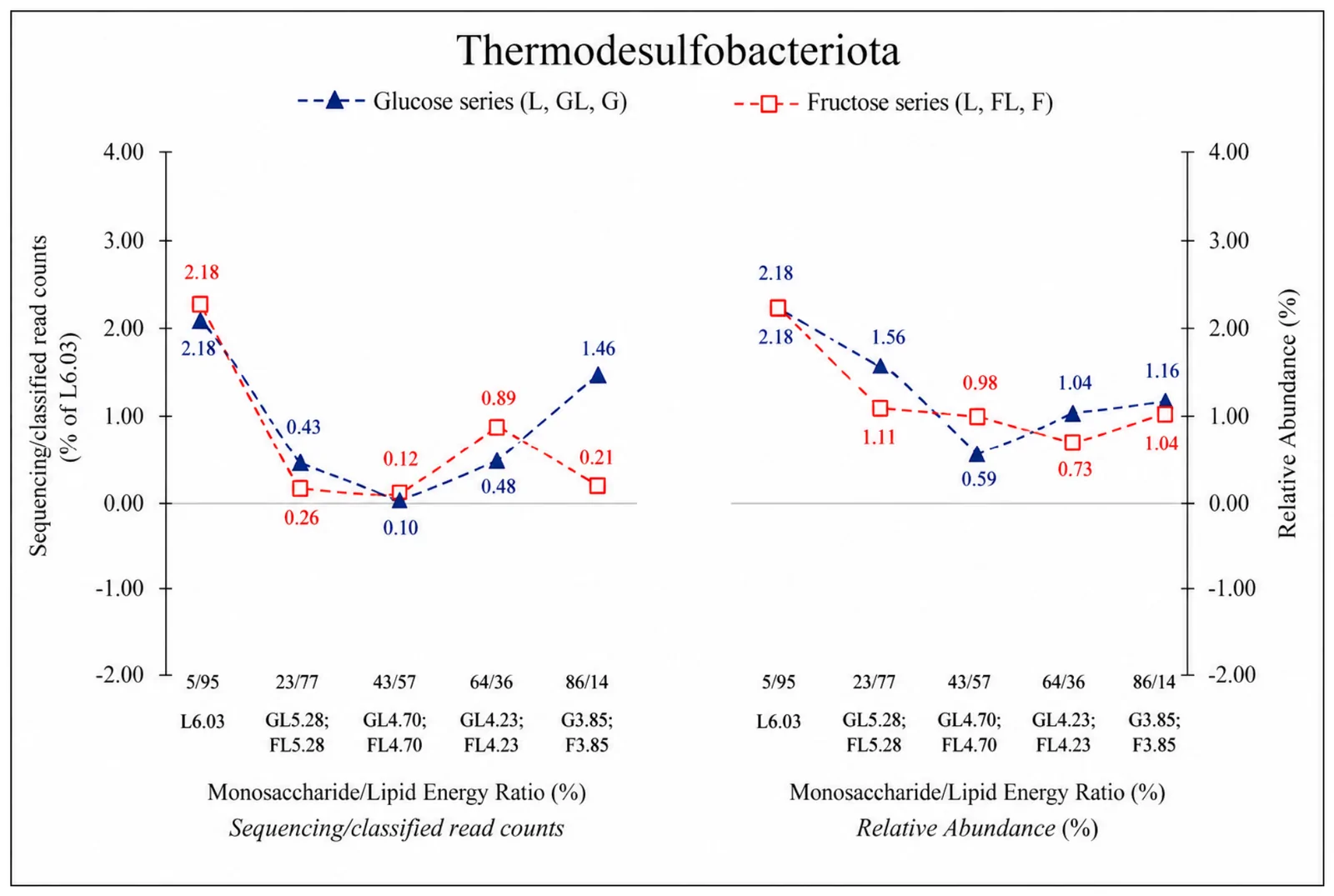
Treatment-level sequencing/classified read counts and relative abundance of Thermodesulfobacteriota across the glucose- and fructose-containing dietary series. Read-count values are expressed relative to the L_6.03_ reference value (L_6.03_ = 0%). Each point represents one pooled cecal microbiome sample; connecting lines are for visual guidance only. Read counts are sequencing-derived and do not represent bacterial abundance. Abbreviations: L, lard; GL, glucose + lard; G, glucose; FL, fructose + lard; F, fructose.

Thermodesulfobacteriota include sulfate-reducing members of the gut microbiota. Phylum-level CR and RA responses across the glucose and fructose dietary series are presented in Figure 11.

Classified read counts: CRs showed a non-linear pattern across the dietary series (Figure 11). Relative to the common L_6.03_ reference normalization, CRs decreased in the mixed lard–monosaccharide diets in both series. In the glucose series, the lowest value occurred at the 43/57 energy ratio (GL_4.70_; 0.10%), followed by an increase to 1.46% at the lard-free endpoint (G_3.85_). In the fructose series, CRs remained low across the mixed diets and declined to 0.21% at the lard-free endpoint (F_3.85_).

Relative abundance: Thermodesulfobacteriota RA also showed a non-linear pattern. From 2.18% in L_6.03_, RA decreased in all mixed lard–monosaccharide diets, reaching 0.59–1.56% in the glucose series and remaining below the control throughout the fructose series. At the lard-free endpoints, RA was 1.16% in G_3.85_ and 1.04% in F_3.85_.

Integrated interpretation: CRs and RA both indicated reduced representation of Thermodesulfobacteriota relative to L_6.03_ across the mixed lard–monosaccharide diets, but their subsequent patterns were not fully concordant. In the glucose series, CRs increased markedly toward the lard-free endpoint, whereas in the fructose series it declined further at F_3.85_. RA remained below the L_6.03_ level throughout both series and showed only partial recovery at the lard-free endpoints. Thus, the relationship between CRs and RA varied across the dietary gradient, with both measures distinguishing G_3.85_ from F_3.85_. Given the reported ability of *Bilophila wadsworthia* to aggravate high-fat-diet-induced metabolic dysfunctions in mice [48], the reduction observed here in the mixed diets further emphasizes the dependence of these taxonomic patterns on dietary context.

**Figure 12:**
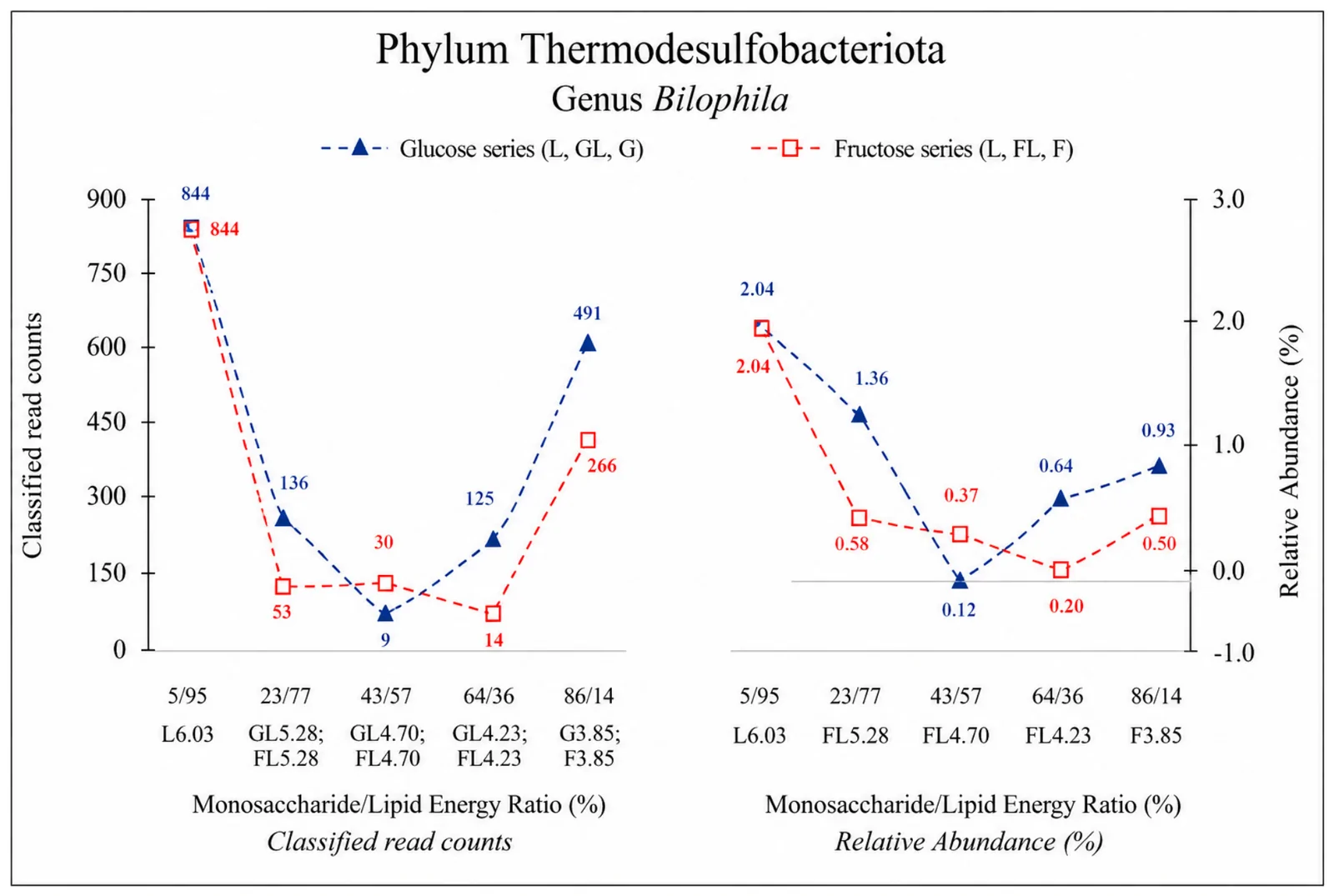
Treatment-level sequencing/classified read counts and relative abundance of *Bilophila* (phylum Thermodesulfobacteriota) across the glucose- and fructose-containing dietary series. Genus-level CR values represent raw, non-normalized classified read counts. Each point represents one pooled cecal microbiome sample; connecting lines are for visual guidance only. Read counts are sequencing-derived and do not represent bacterial abundance. Abbreviations: L, lard; GL, glucose + lard; G, glucose; FL, fructose + lard; F, fructose.

At the genus level, the response was further examined in *Bilophila* (Figure 12), a member of Thermodesulfobacteriota. *Bilophila wadsworthia* has been shown to aggravate high-fat-diet-induced metabolic dysfunctions in mice [48], providing a biologically relevant context for examining the dietary response of this genus.

Classified read counts: CRs showed a pronounced non-linear pattern (Figure 12). From 844 reads in L_6.03_, CRs declined sharply in the mixed lard–monosaccharide diets, reaching minimum values of 9 reads in GL_4.70_ and 14 reads in FL_4.23_, corresponding to reductions of 98.9% and 98.3%, respectively. CRs subsequently increased as dietary lard was further replaced, reaching 491 reads in G_3.85_ and 266 reads in F_3.85_.

Relative abundance: RA showed a broadly concordant non-linear pattern. From 2.04% in L_6.03_, RA declined markedly in the mixed diets and subsequently increased toward the lard-free endpoints, reaching 0.93% in G_3.85_ and 0.50% in F_3.85_.

Integrated interpretation: CRs and RA showed broadly concordant patterns for *Bilophila*, with marked reductions in the mixed lard–monosaccharide diets followed by partial recovery toward the lard-free endpoints. The recovery was more pronounced at the glucose than at the fructose endpoint in both data representations. Consistent with the reported association of *Bilophila* with high-fat diets and bile acid metabolism [48], the highest values occurred in the carbohydrate-free, lard-rich control. However, the partial recovery toward the lard-free endpoints indicates that the response did not simply follow dietary lard content. Rather, the pronounced non-linear pattern suggests an association with the broader lard–monosaccharide dietary context.

### 3.9. Phylum Verrucomicrobiota

**Figure 13:**
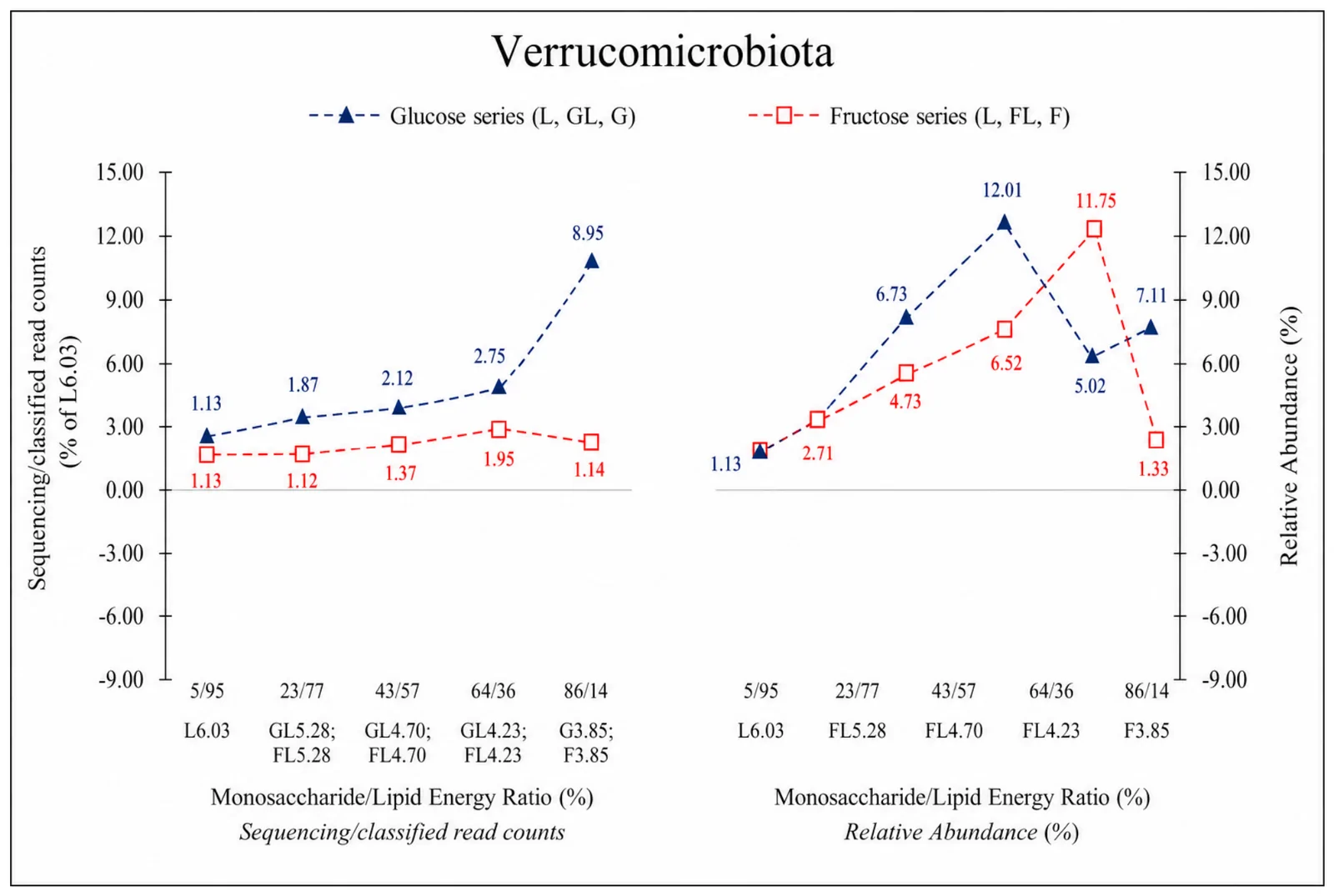
Treatment-level sequencing/classified read counts and relative abundance of Verrucomicrobiota across the glucose- and fructose-containing dietary series. Read-count values are expressed relative to the mean value of the L_6.03_ reference group (L_6.03_ = 0%). Each point represents one pooled cecal microbiome sample; connecting lines are for visual guidance only. Abbreviations: L, lard; GL, glucose + lard; G, glucose; FL, fructose + lard; F, fructose.

Verrucomicrobiota represents a relatively minor component of the gut microbiota in the present study. Sequencing/classified read counts and relative abundance for this phylum across the glucose and fructose dietary series are presented in Figure 13.

Classified read counts: Verrucomicrobiota CRs showed distinct non-linear patterns in the two dietary series (Figure 13). In the glucose series, CRs showed an overall increase as dietary lard was progressively replaced, rising from 1.13 relative to the L_6.03_ reference to 1.87, 2.12, and 2.75 in the mixed diets and reaching 8.95 at the lard-free endpoint G_3.85_. In the fructose series, the corresponding values showed smaller changes, increasing from 1.13 in L_6.03_ to 1.12, 1.37, and 1.95 in the mixed diets before declining to 1.14 at F_3.85_. Thus, the greatest divergence between the two series occurred under lard-free conditions.

Relative abundance: Verrucomicrobiota RA showed a broadly similar glucose–fructose divergence (Figure 13). From 1.13% in L_6.03_, RA increased in the glucose series to 2.71%, 6.73%, and 12.01% across the mixed diets and remained elevated at 7.11% in G_3.85_. In the fructose series, RA also increased across the mixed diets, reaching 2.71%, 4.73%, 6.52%, and 11.75%, but declined markedly to 1.33% at F_3.85_. Thus, the greatest divergence between the two series again occurred under lard-free conditions.

Integrated interpretation: CRs and RA showed broadly concordant dietary patterns for Verrucomicrobiota, although their magnitude varied across treatments. Both measures distinguished the glucose and fructose series most clearly under lard-free conditions, where Verrucomicrobiota remained markedly higher in G_3.85_ than in F_3.85_. This contrasting endpoint response suggests that differences associated with monosaccharide identity became more apparent as dietary lard was reduced.

Given the mucin-utilizing capacity of *Akkermansia muciniphila* [49,50,51] and reports of microbial alterations associated with high-fructose diets [52], the contrasting glucose and fructose endpoint profiles are consistent with a strong dependence of this phylum on dietary substrate context.

At the genus level, the Verrucomicrobiota response was further examined through *Akkermansia,* particularly *Akkermansia muciniphila*, which has been widely associated with intestinal and metabolic health [49,50,53,54]. Altered *Akkermansia* abundance has also been reported in obesity, diabetes, inflammatory bowel disease, and colorectal cancer, although these associations are context-dependent [53,54,55,56]. *Akkermansia muciniphila* is specialized for the utilization of host-derived mucin as a carbon and energy source [49,50,51].

**Figure 14:**
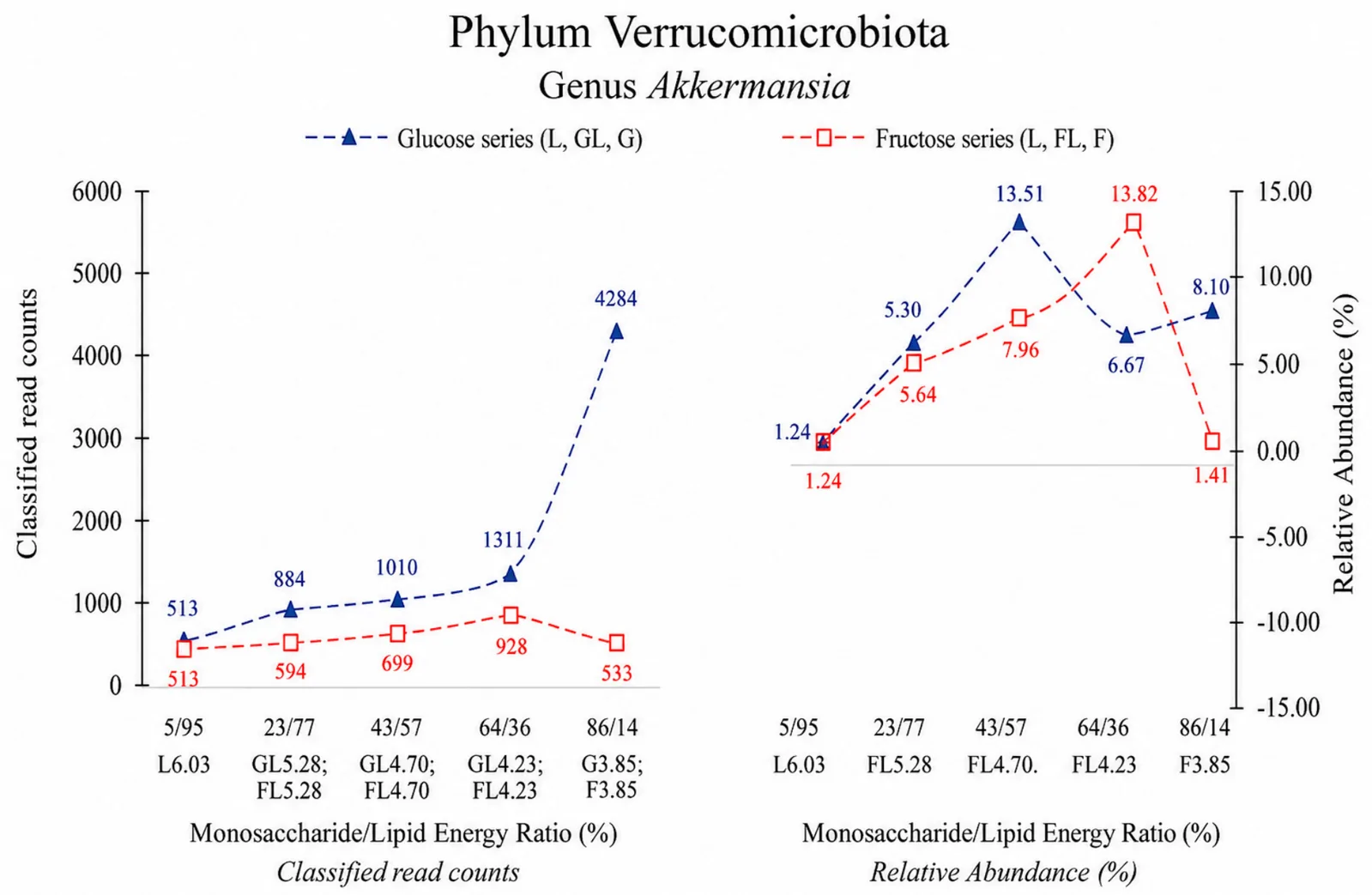
Treatment-level sequencing/classified read counts and relative abundance of *Akkermansia* (phylum Verrucomicrobiota) across the glucose- and fructose-containing dietary series. Genus-level CR values represent raw, non-normalized classified read counts. Each point represents one pooled cecal microbiome sample; connecting lines are for visual guidance only. Abbreviations: L, lard; GL, glucose + lard; G, glucose; FL, fructose + lard; F, fructose.

High-fructose diets have also been associated with reduced microbial richness and depletion of beneficial gut microorganisms [52]. The genus-level response of *Akkermansia* is presented in Figure 14.

Classified read counts: *Akkermansia* CRs increased progressively in the glucose series, from 513 reads in L_6.03_ to 884–1311 reads in the mixed diets and 4284 reads in G_3.85_, approximately 8.3-fold above the control. In contrast, CRs showed smaller changes in the fructose series, ranging from 594 to 928 reads in the mixed diets and reaching 533 reads in F_3.85_.

Relative abundance: *Akkermansia* RA showed a partly different pattern. In the glucose series, RA increased from 1.24% in L_6.03_ to a maximum of 13.51% in GL_4.70_ and remained elevated at 8.10% in G_3.85_. In the fructose series, RA increased across the mixed diets, reaching a maximum of 13.82% in FL_4.23_, but declined sharply to 1.41% in F_3.85_.

Integrated interpretation: The combined evaluation of sequencing/classified read counts and relative abundance revealed an important distinction in the *Akkermansia* response. In the glucose series, increasing sequencing/classified read counts were accompanied by an overall increase in relative abundance, culminating in the markedly elevated G_3.85_ endpoint. In the fructose series, however, the high relative abundance observed in the mixed diets was not accompanied by a comparable increase in sequencing/classified read counts. This indicates that the pronounced increase in the proportional representation of *Akkermansia* in these groups was at least partly associated with changes in the proportional structure of the overall microbial community. The particularly strong divergence at the lard-free endpoint was evident in both measures, with substantially higher *Akkermansia* values in G_3.85_ than in F_3.85_.

### 3.10. Hierarchical Clustering

**Figure 15:**
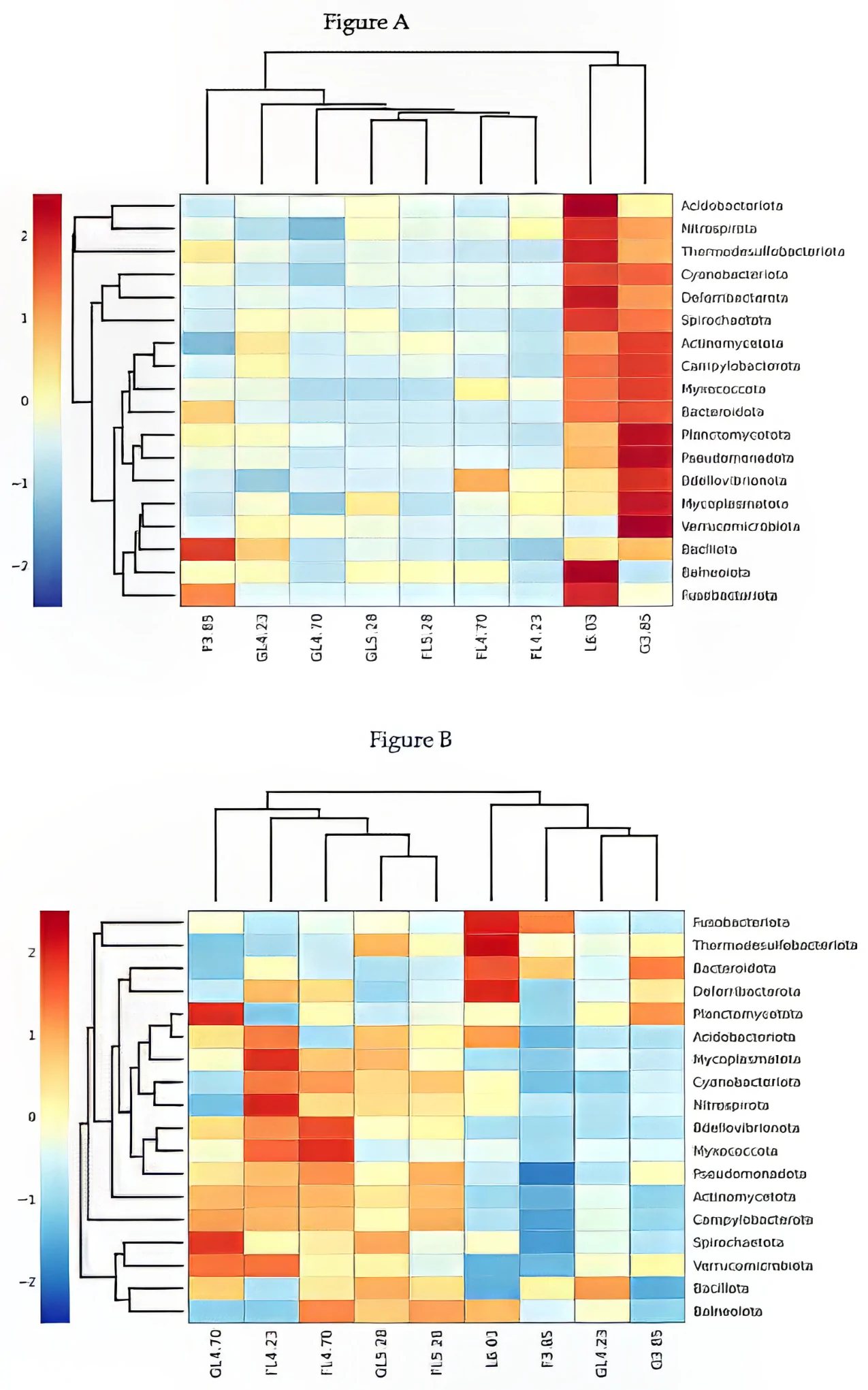
Two-way hierarchical clustering of phylum profiles across the nine dietary treatments. Heat-map values were standardized as z-scores, with blue and red indicating relatively lower and higher standardized values, respectively. (**A**) Clustering based on sequencing/classified read counts. (**B**) Clustering based on relative abundance. Bacterial phyla (rows) and dietary treatments (columns) were hierarchically clustered. Each treatment profile represents a single pooled cecal microbiome sample; therefore, the clustering is descriptive and does not estimate within-group variability. Abbreviations: L, lard; GL, glucose + lard; G, glucose; FL, fructose + lard; F, fructose.

Hierarchical clustering was performed to evaluate the overall associations of dietary treatments with cecal microbial community organization. Unlike the analyses of individual bacterial phyla, hierarchical clustering evaluates the microbiota as an integrated community, allowing coordinated patterns of community organization to be identified across both dietary treatments and bacterial phyla. Cluster analyses were performed independently using sequencing/classified read counts and relative abundance data at the phylum level (Figure 15), enabling comparison of the observed clustering patterns across the two sequencing-derived representations.

Classified read counts: Hierarchical clustering based on sequencing/classified read counts revealed a distinct separation of the L_6.03_ and G_3.85_ groups from the remaining dietary treatments (Figure 15A). These two dietary endpoints formed a separate major cluster, whereas F_3.85_ clustered with the mixed lard–monosaccharide diets. Within the larger cluster, several glucose- and fructose-containing treatments showed close relationships, but no consistent separation according to monosaccharide identity was evident. Thus, the clustering pattern was non-linear and could not be explained by either dietary lard content or monosaccharide identity alone.

Relative abundance: Hierarchical clustering based on relative abundance produced a different detailed arrangement of the dietary treatments (Figure 15B). Two major treatment clusters were again apparent, but their composition differed from that obtained using sequencing/classified read counts. Several mixed lard–monosaccharide diets clustered together, whereas L_6.03_ grouped with a subset of the higher-monosaccharide treatments. Notably, G_3.85_ clustered closely with GL_4.23_ rather than forming an isolated endpoint branch. Thus, although the precise relationships among treatments depended on the abundance measure, both analyses demonstrated structured and non-linear treatment-associated differences in microbial community composition.

Comparison of glucose- and fructose-containing diets: Neither clustering approach produced a consistent separation of the glucose- and fructose-containing dietary series. Instead, treatments from the two series were interspersed within the major clusters, indicating that monosaccharide identity alone was not the principal determinant of phylum-level community organization. At the same time, differences between corresponding glucose- and fructose-containing treatments were evident at several dietary compositions, demonstrating that monosaccharide identity contributed to the overall community pattern in a diet-dependent manner.

Integrated interpretation: Hierarchical clustering based on sequencing/classified read counts and relative abundance revealed related but not identical patterns of treatment-associated community organization [22,23,24,25,26]. Both analyses demonstrated structured differences among dietary treatments and the absence of a simple separation according to monosaccharide identity, whereas the precise positioning of individual dietary groups differed between the two data representations. Because sequencing/classified read counts retain information on differences in detectable taxonomic reads, whereas relative abundance describes the proportional composition of the classified microbial community, the differences between the two dendrograms are consistent with the complementary information provided by these measures.

Collectively, hierarchical clustering demonstrated a complex, non-linear relationship between dietary composition and cecal microbial community organization. Neither progressive replacement of dietary lard nor monosaccharide identity alone accounted for the observed clustering patterns. Rather, community organization reflected the combined dietary context, with the relative contributions of lard content and monosaccharide identity varying across dietary compositions.

### 3.11. Network Analysis

To explore coordinated changes among bacterial taxa along the two dietary series, taxon–taxon Pearson correlation networks were constructed separately for the glucose and fructose series using Konf00-normalized treatment-level taxonomic data. The networks were used to characterize patterns of taxonomic co-variation across the dietary gradients rather than to infer direct ecological or causal interactions [10,57]. Because each network was based on five treatment-level profiles within a dietary series, the analysis was considered exploratory and hypothesis-generating.

**Figure 16:**
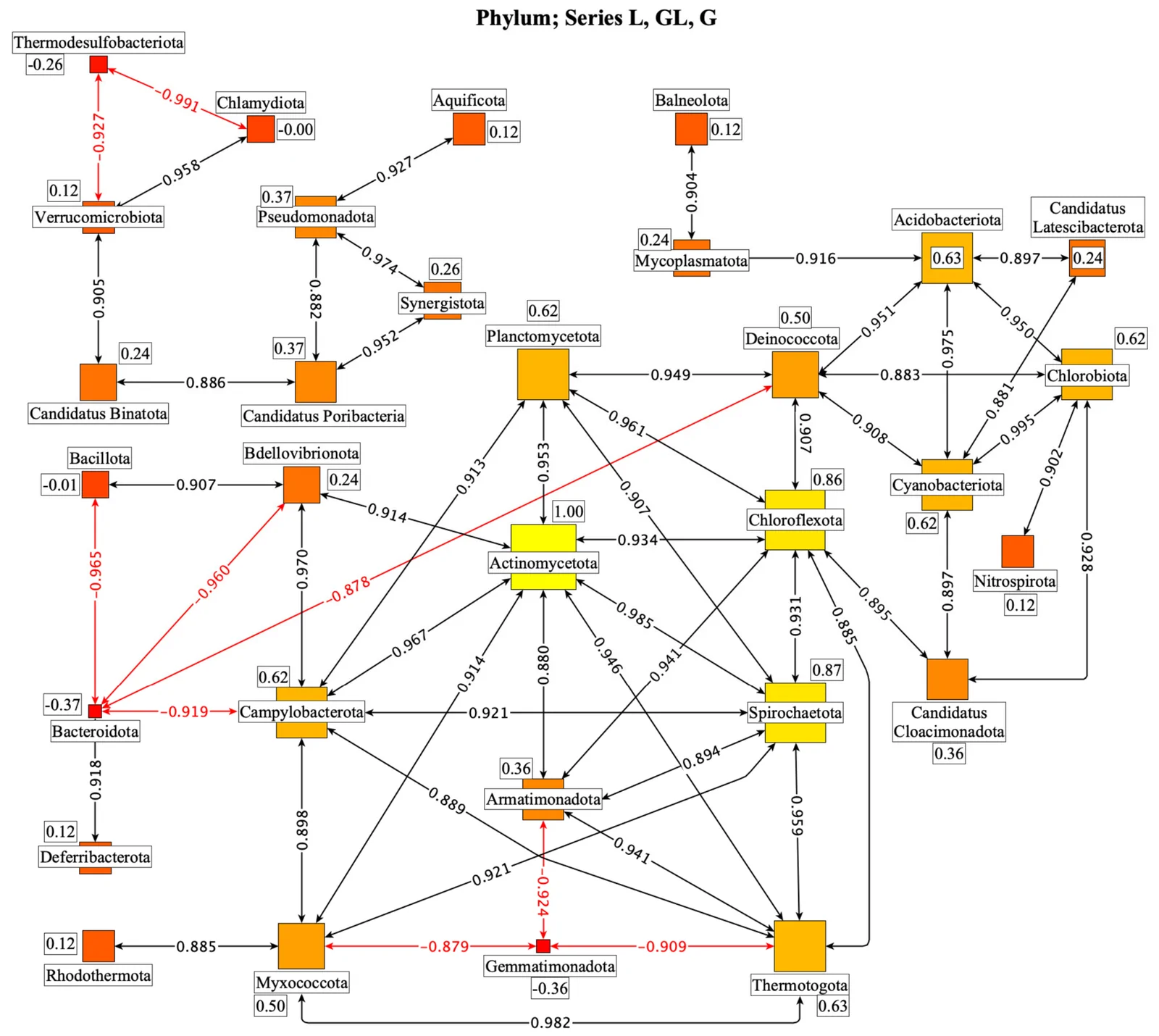
Phylum-level correlation network of the glucose dietary series (L, GL, G). The network was constructed from Konf00-normalized treatment-level taxonomic data across the five dietary treatments of the glucose series (*n* = 5 treatment-level observations; df = 3). Pearson correlations meeting the two-sided *p* < 0.05 criterion (|r| ≥ 0.878) were retained. Taxa were additionally filtered according to their maximum relative abundance within the glucose dietary series using a threshold of ≥0.2%. Only taxa participating in at least one retained correlation after abundance filtering are displayed. Black edges indicate positive and red edges negative correlations; numerical labels show Pearson’s r. Node background color follows network centrality, ranging from yellow to red with increasing centrality; numerical values adjacent to genera indicate the corresponding network-centrality values.

Glucose series—phylum level: The glucose-series phylum network showed an extensively interconnected structure containing both positive and negative correlations (Figure 16). Actinomycetota occupied the most central position (centrality = 1.00). Other relatively high-centrality taxa included Spirochaetota (0.87) and Chloroflexota (0.86), while Campylobacterota, Planctomycetota, Cyanobacteriota, and Chlorobiota each had a centrality value of 0.62.

Most retained associations were positive, but several strong negative correlations were also present. Bacteroidota was negatively correlated with Bacillota (r = −0.965), Campylobacterota (r = −0.919), and Bdellovibrionota (r = −0.960). Gemmatimonadota showed negative correlations with Armatimonadota (r = −0.924), Myxococcota (r = −0.879), and Thermotogota (r = −0.909). Thermodesulfobacteriota was negatively associated with Verrucomicrobiota (r = −0.927) and Chlamydiota (r = −0.991). These negative associations occurred within a broader network dominated by positive co-variation.

**Figure 17:**
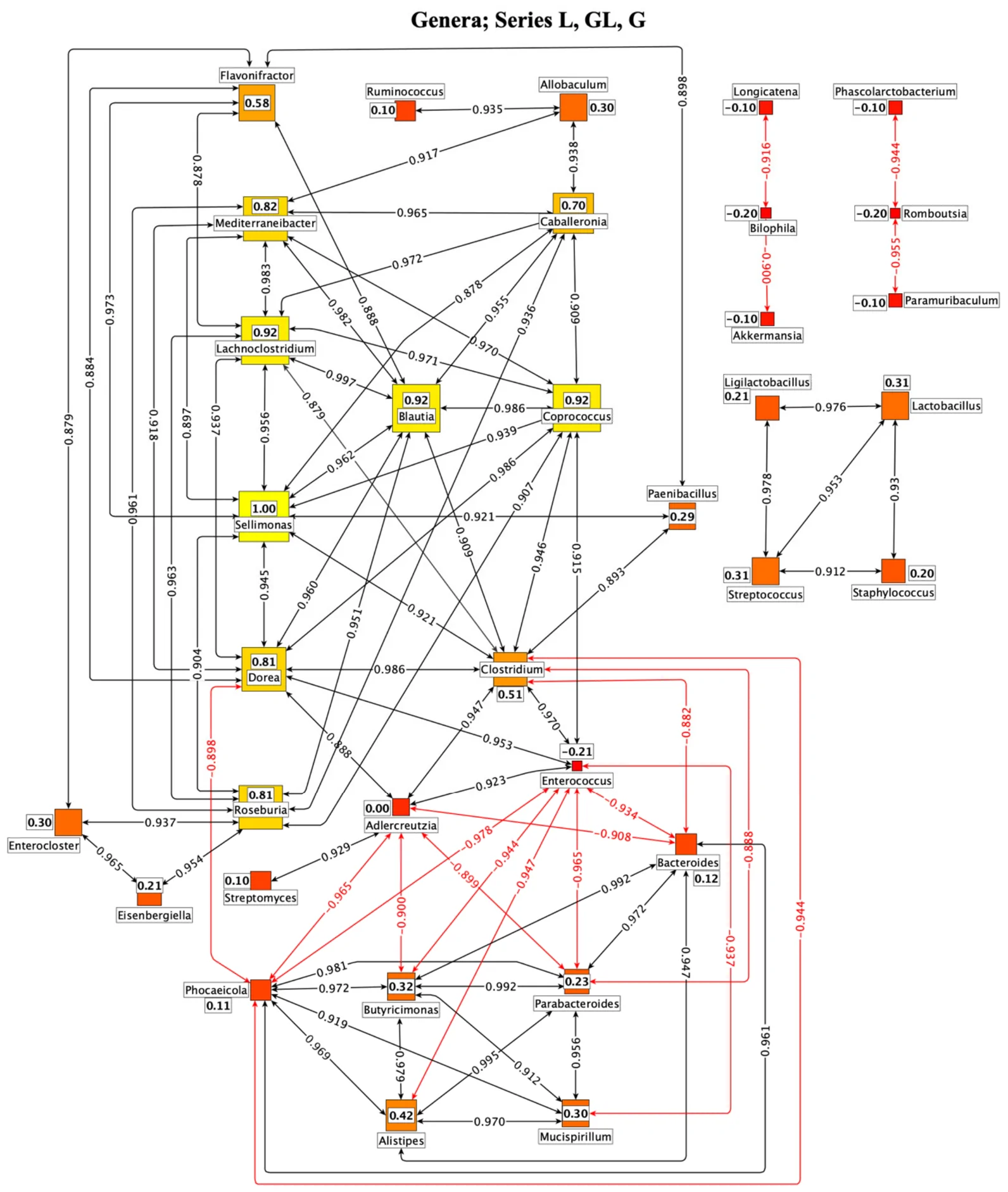
Genus-level correlation network of the glucose dietary series (L, GL, and G). The network was constructed from Konf00-normalized treatment-level data (*n* = 5; df = 3). Pearson correlations with |r| ≥ 0.878 (*p* < 0.05) were retained, and genera were filtered by a maximum relative abundance of ≥0.2% within the series. Only genera participating in at least one retained correlation are shown. Black and red edges indicate positive and negative correlations, respectively; edge labels show Pearson’s r. Node background color indicates network centrality, ranging from red to yellow with increasing centrality; numerical values indicate the corresponding centrality values.

Glucose series—genus level: The glucose-series genus network was denser than the corresponding phylum-level network and contained a large, highly interconnected central component together with several smaller peripheral structures (Figure 17).

*Sellimonas* occupied the most central position (centrality = 1.00). Other genera with relatively high centrality included *Blautia, Coprococcus,* and *Lachnoclostridium* (0.92); *Mediterraneibacter* (0.82); *Dorea* and *Roseburia* (0.81); *Caballeronia* (0.70); *Flavonifractor* (0.58); and *Clostridium* (0.51).

Positive correlations predominated within the central component, while several strong negative associations occurred elsewhere in the network. These included *Adlercreutzia–Butyricimonas* (r = −0.900), *Adlercreutzia–Parabacteroides* (r = −0.909), *Enterococcus–Bacteroides* (r = −0.908), *Enterococcus–Parabacteroides* (r = −0.957), and *Lachnoclostridium–Clostridium* (r = −0.983). Smaller peripheral structures included the negatively connected *Longicatena–Bilophila–Akkermansia* and *Phascolarctobacterium–Romboutsia–Paramuribaculum* chains, whereas *Ligilactobacillus, Lactobacillus, Streptococcus*, and *Staphylococcus* formed a positively correlated component.

**Figure 18:**
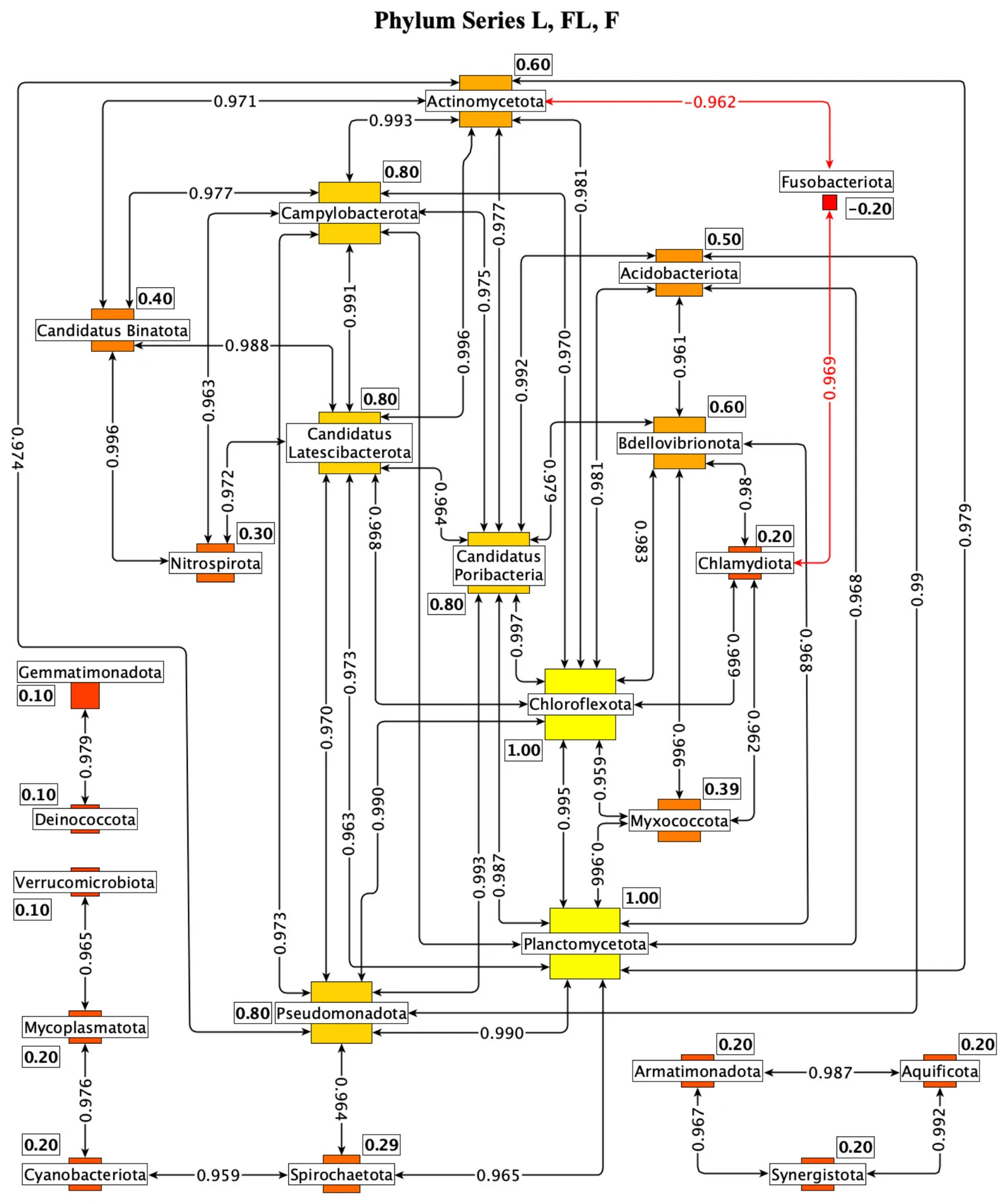
Phylum-level correlation network of the fructose dietary series (L, FL, and F). The network was constructed from Konf00-normalized treatment-level data (*n* = 5; df = 3). Pearson correlations with |r| ≥ 0.878 (*p* < 0.05) were retained, and taxa were filtered by a maximum relative abundance of ≥0.2% within the series. Only taxa participating in at least one retained correlation are shown. Black and red edges indicate positive and negative correlations, respectively; edge labels show Pearson’s r. Node background color indicates network centrality, ranging from red to yellow with increasing centrality; numerical values indicate the corresponding centrality values.

Fructose series—phylum level: The fructose-series phylum network was also extensively interconnected but was dominated by positive correlations (Figure 18). Planctomycetota and Chloroflexota shared the highest centrality value (1.00), while Pseudomonadota (0.80) also occupied a relatively central position within the network.

Strong positive associations included Actinomycetota–Campylobacterota (r = 0.993), Candidatus Binatota–Candidatus Latescibacterota (r = 0.988), Planctomycetota–Pseudomonadota (r = 0.990), and Armatimonadota–Aquificota (r = 0.987). Negative co-variation was more localized and included Fusobacteriota–Actinomycetota (r = −0.962) and Chlamydiota–Fusobacteriota (r = −0.969).

**Figure 19:**
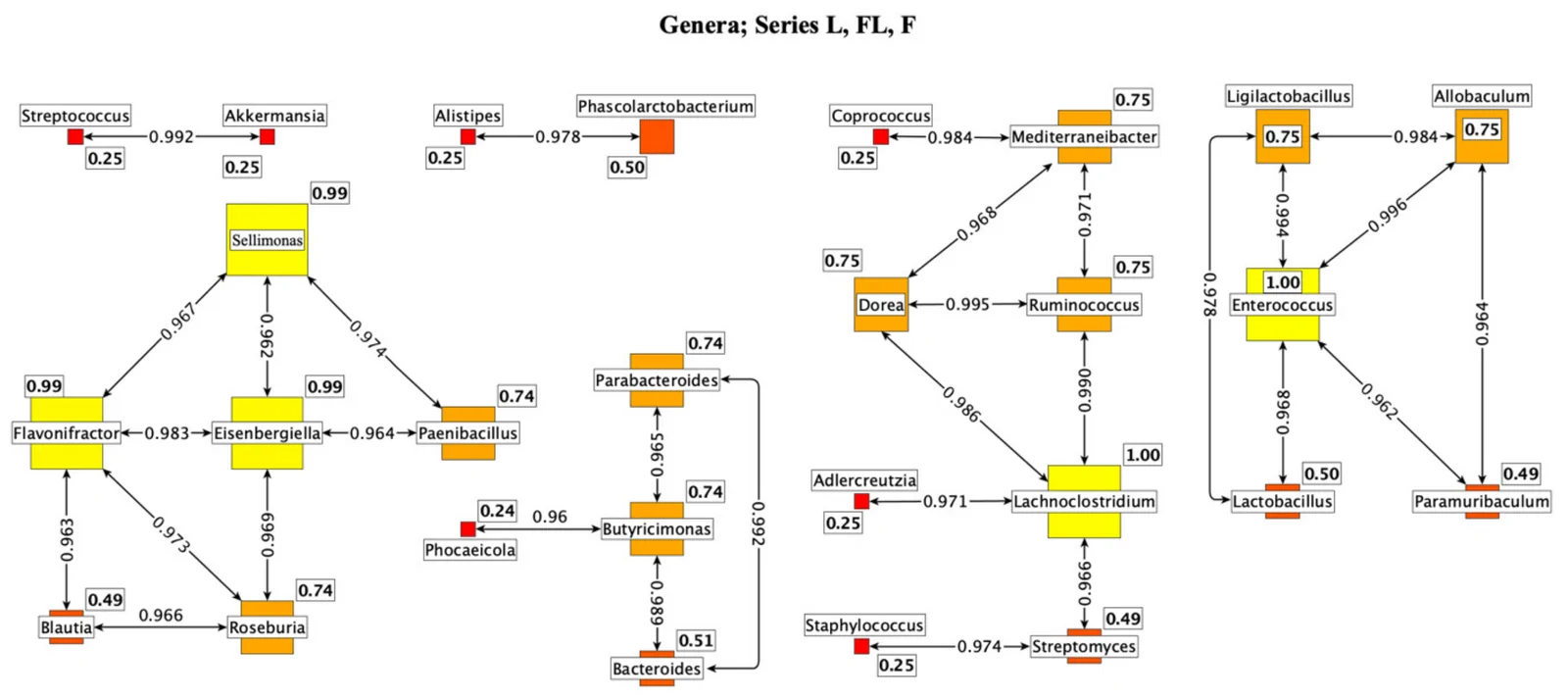
Genus-level correlation network of the fructose dietary series (L, FL, and F). The network was constructed from Konf00-normalized treatment-level taxonomic data across the five dietary treatments of the fructose series (*n* = 5 treatment-level observations; df = 3). Pearson correlations meeting the two-sided *p* < 0.05 criterion (|r| ≥ 0.878) were retained. Genera were additionally filtered according to their maximum relative abundance within the fructose dietary series using a threshold of ≥1.0%. Only genera participating in at least one retained correlation after abundance filtering are displayed. Black edges indicate positive and red edges negative correlations; numerical labels show Pearson’s r. Node background color follows network centrality, ranging from yellow to red with increasing centrality; numerical values adjacent to genera indicate the corresponding network-centrality values.

Fructose series—genus level: The fructose-series genus network was more compartmentalized than the corresponding glucose-series network, with several interconnected groups rather than one dominant central component (Figure 19).

The highest centrality value (1.00) was shared by *Enterococcus* and *Lachnoclostridium*. *Dorea*, *Ruminococcus*, *Mediterraneibacter*, *Ligilactobacillus*, and *Allobaculum* had centrality values of 0.75, while *Paenibacillus*, *Parabacteroides*, *Butyricimonas*, and *Roseburia* had centrality values of 0.74.

Strong positive correlations included *Streptococcus–Akkermansia* (r = 0.992), *Flavonifractor–Eisenbergiella* (r = 0.983), *Dorea–Ruminococcus* (r = 0.995), *Ruminococcus–Lachnoclostridium* (r = 0.990), *Enterococcus–Allobaculum* (r = 0.996), and *Enterococcus–Ligilactobacillus* (r = 0.994). In contrast to the glucose-series genus network, the fructose-series genus network was characterized almost entirely by positive correlations distributed among several interconnected components.

Integrated interpretation: The network analysis adds a community-level dimension to the taxonomic results by showing how taxon responses were organized along the dietary gradients.

Network centrality should be distinguished from quantitative taxonomic dominance: it reflects how strongly a taxon is embedded in the pattern of coordinated treatment-level variation rather than its numerical representation in the community.

This distinction was particularly evident for *Planctomycetota,* which shared the highest centrality value in the fructose phylum network (centrality = 1.00) despite being represented by only 1–10 classified reads across treatments. At such low representation, high centrality alone provides no indication of a quantitatively important effect on the host.

The dietary gradient provides the common experimental axis along which these coordinated taxonomic responses were observed. Digested and absorbed nutrients influence host metabolism and physiology, while the gastrointestinal microbiota is exposed to diet-derived substrates and digestion products throughout their passage along the gastrointestinal tract.

The cecal microbiota examined here therefore represents a distal, integrated outcome of this dietary exposure and of the microbial ecological and metabolic processes developing along the gastrointestinal tract. Diet may select taxa according to substrate availability and local environmental conditions, while resulting changes in microbial metabolism may further modify the environment experienced by other community members [58,59].

Accordingly, parallel taxonomic trajectories may reflect similar responses to the nutritional environment or secondary metabolic relationships such as cross-feeding, whereas opposing trajectories may reflect differential dietary selection, competition for substrates, or metabolite-mediated changes in the microbial environment [59,60].

The present correlations cannot distinguish among these possibilities or establish direct microbial interactions. Nevertheless, secondary microbial relationships, where present, may themselves form part of the broader community response associated with the dietary gradient.

Rather than defining a hierarchy of dominant microorganisms, the networks therefore describe how the cecal microbial community was organized along the two dietary gradients. The microbiome response to dietary composition can thus be viewed at complementary levels: as changes in individual taxa and as coordinated community-level co-variation in which primary responses to dietary conditions may be accompanied or propagated by secondary microbial relationships.

### 3.12. Humoral Immune Response

**Figure 20:**
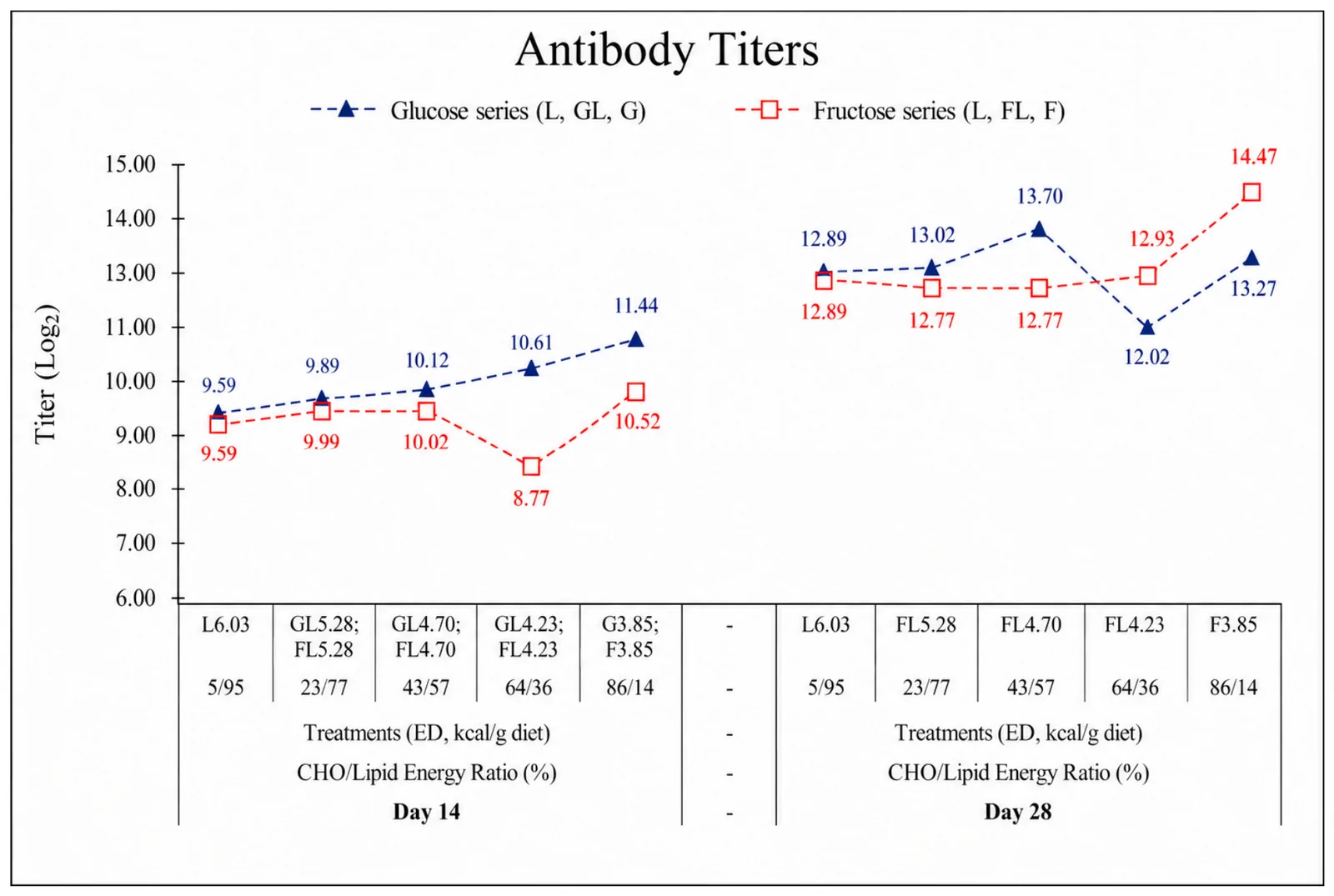
Serum anti-ovalbumin (anti-OVA) antibody titers on Days 14 and 28 in rats fed the glucose- and fructose-containing dietary series. Values are group means (*n* = 8 per dietary treatment) expressed as log_2_ titers; connecting lines are for visual guidance only. Abbreviations: OVA, ovalbumin; L, lard; GL, glucose + lard; G, glucose; FL, fructose + lard; F, fructose; CHO, carbohydrate; ED, energy density.

The humoral immune response following ovalbumin immunization was evaluated as an independently replicated host outcome (*n* = 8 per dietary treatment) to determine whether dietary composition was accompanied by differences in adaptive immune responsiveness (Figure 20).

Humoral immunity is influenced by nutritional status and may vary with dietary composition [61,62,63,64]. In the present study, anti-OVA responses were measured in individual animals and are interpreted as host-response outcomes separate from the pooled treatment-level microbiome observations; the design does not establish a microbiota–immune causal relationship.

Primary immune response: Antibody titers increased in all dietary groups by Day 14 following primary immunization. In the glucose-containing dietary series, antibody titers increased progressively as dietary glucose content increased and dietary lard was reduced. In contrast, fructose-containing diets were associated with a more variable response, with no consistent pattern across the dietary gradient.

Secondary immune response: Booster immunization resulted in a marked increase in antibody titers in all groups by Day 28, consistent with the expected secondary humoral response. However, the dietary patterns differed from those observed during the primary response. In the fructose-containing dietary series, antibody titers increased progressively as dietary fructose content increased and dietary lard was reduced, reaching their highest values in the lard-free F_3.85_ group. In contrast, the glucose-containing dietary series exhibited a more moderate secondary response.

Comparison of glucose- and fructose-associated humoral immune responses: The observed antibody trajectories differed descriptively between the glucose- and fructose-containing dietary series. The glucose series showed a progressive pattern in the primary response, whereas the fructose series showed a more pronounced increase after booster immunization. These patterns are hypothesis-generating and should not be interpreted as evidence of a specific nutrient-mediated immune mechanism.

Overall, the primary and secondary humoral immune responses showed different descriptive patterns across the dietary treatments and between the glucose- and fructose-containing dietary series. Because these measurements were obtained from individual animals, they provide a replicated host-response outcome. Although treatment-associated microbiome differences were observed in parallel, the present design does not test or establish a microbiota–immune mechanism; any such relationship remains a hypothesis for future studies with independently sequenced biological replicates.

### 3.13. Cross-Representation Co-Variation of Classified Reads and Relative Abundance

To evaluate how data representation influenced the observed treatment responses, original non-normalized phylum-level classified-read (CR) profiles were compared with the corresponding relative-abundance (RA) profiles within the glucose and fructose dietary series. Pearson correlation networks were constructed across the five treatment-level profiles per series (*n* = 5; df = 3), retaining CR–RA associations with |*r*| ≥ 0.878 (*p* < 0.05). No correction for multiple comparisons was applied; therefore, these networks were interpreted as descriptive, hypothesis-generating representations of treatment-level co-variation.

**Figure 21:**
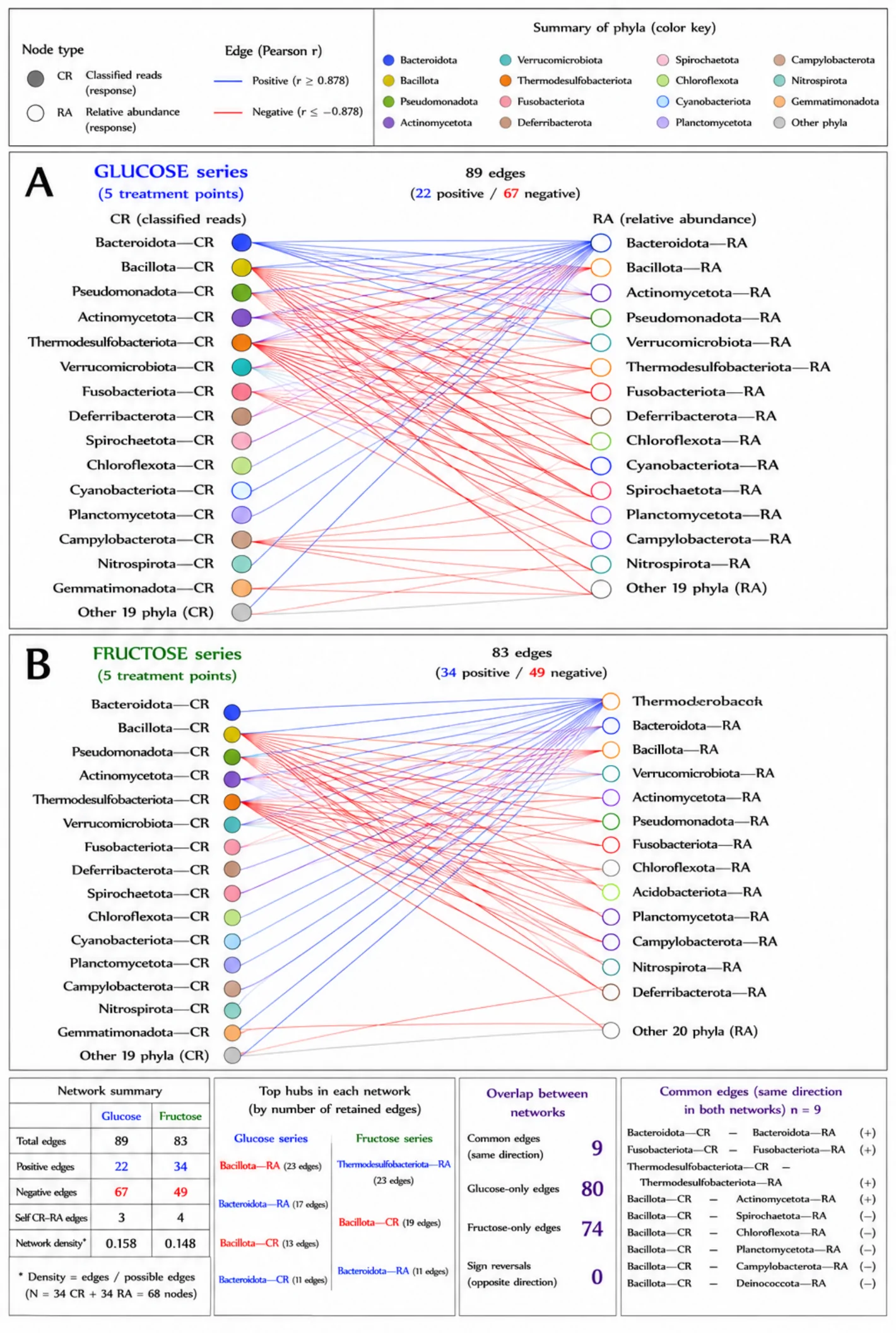
Cross-representation co-variation networks between phylum-level classified-read (CR) and relative-abundance (RA) profiles in the glucose (**A**) and fructose (**B**) dietary series. Networks were based on Pearson correlations across five treatment-level profiles per series (*n* = 5; df = 3), retaining associations with |r| ≥ 0.878 (*p* < 0.05). Blue and red edges indicate positive and negative correlations, respectively; filled and open nodes represent CR and RA profiles. Networks describe treatment-level co-variation and do not imply direct ecological interactions. Abbreviations: CR, classified reads; RA, relative abundance; L, lard; GL, glucose + lard; G, glucose; FL, fructose + lard; F, fructose.

Overall, 89 CR–RA associations were retained in the glucose series (22 positive and 67 negative) and 83 in the fructose series (34 positive and 49 negative) (Figure 21). Negative associations therefore predominated particularly in the glucose network, whereas the fructose network showed a more balanced distribution. The glucose network was characterized by a predominantly negative co-variation structure centered on Bacillota-RA, together with a prominent positive Bacteroidota-RA-associated module. In contrast, the fructose network showed a different organization, with Thermodesulfobacteriota-RA as a major connectivity hub (23 retained CR associations) and Bacillota-CR forming another prominent component (19 retained associations, many negative).

Despite similar numbers of retained associations, only nine CR–RA relationships were shared between the glucose and fructose networks, all with the same direction of association. Moreover, only three same-phylum CR–RA associations were retained in the glucose series (Bacteroidota, Deferribacterota, and Fusobacteriota) and four in the fructose series (Bacteroidota, Fusobacteriota, Thermodesulfobacteriota, and Verrucomicrobiota). Thus, most associations linked the CR response of one phylum with the RA response of another, indicating that the two sequencing-derived representations captured substantially different patterns of treatment-level co-variation.

Although CRs and RA are mathematically related, their treatment-response profiles could be concordant, opposite in direction, or markedly different in shape. Because RA is calculated as the taxon-specific classified-read count relative to the total classified-read pool, variation in either component can alter the proportional representation of individual taxa. In the absence of an internal quantitative standard or an independent measurement of total microbial load, the extent to which variation in total CRs reflects biological versus technical factors cannot be determined. Consequently, differences between CR- and RA-based profiles should be interpreted as representation-dependent features of the sequencing dataset rather than as evidence of changes in absolute microbial abundance.

Joint evaluation of CRs and RA therefore provides complementary information by identifying treatment-response patterns that are concordant across the two sequencing-derived representations and those that are strongly representation-dependent. Neither representation, however, provides a measure of absolute bacterial abundance in the absence of quantitative calibration.

### 3.14. Limitations and Future Perspectives

The use of pooled cecal samples (one pooled sample per dietary group, comprising samples from *n* = 8 animals) precluded assessment of inter-individual variability and individual-level statistical inference for microbiome outcomes. Accordingly, microbiome findings should be interpreted as treatment-level observations. This limitation does not apply to the humoral immune response, which was evaluated in individual animals (*n* = 8 per dietary group).

Classified read counts are sequencing-derived measurements and do not represent absolute microbial abundance. Without an internal quantitative standard or independent measurement of total bacterial load, they cannot be converted into quantitative bacterial abundance, whereas relative abundance is constrained by the compositional nature of microbiome data [22,23,24,25,26]. The two representations were therefore interpreted as complementary descriptions of the observed sequencing dataset.

Correlation analyses were derived from five treatment-level observations within each monosaccharide series (*n* = 5; df = 3), using nominal screening thresholds and without correction for multiple comparisons. The resulting associations may reflect common responses to the dietary gradient, compositional dependence where relative-abundance data are involved, indirect relationships, or chance co-variation. They are therefore interpreted as exploratory patterns of treatment-level co-variation rather than evidence of direct ecological interactions or causal relationships.

The experimental design nevertheless provides a controlled framework for examining microbial responses to progressive changes in dietary macronutrient composition [65]. Although the present rat data cannot be directly extrapolated to humans, the observed patterns support further investigation of how combinations and relative proportions of dietary fats and simple carbohydrates influence intestinal microbial organization.

Future studies should extend this dietary-gradient approach to individual-animal microbiome profiling combined with quantitative microbial measurements and host physiological, metabolic, and immune responses. Such integrated designs will allow direct investigation of host–microbiota relationships across controlled dietary gradients, providing a basis for determining how diet-associated microbial reorganization relates to host adaptation and function.

## 4. Conclusions

The findings should be interpreted within the methodological and analytical limitations described above. Nevertheless, the controlled dietary-gradient design allowed several consistent features of the microbial response to progressive replacement of dietary lard by glucose or fructose to be identified. Rather than indicating a simple distinction between the two monosaccharides, the results demonstrate that the microbial response depended on the dietary lard–monosaccharide balance, the taxonomic level examined, and the position along the dietary gradient. The principal conclusions are as follows:

1. Microbial responses could not be characterized by a uniform glucose- or fructose-associated pattern; monosaccharide effects were taxon-dependent and varied with the dietary lard–monosaccharide balance.
2. Phylum- and genus-level responses did not necessarily change in parallel, indicating that higher taxonomic levels may conceal substantial genus-specific reorganization.
3. Non-linearity was a recurring feature across taxa: intermediate lard–monosaccharide diets frequently produced distinct microbial patterns rather than simple transitions between the dietary endpoints.
4. Glucose- and fructose-containing diets were also associated with distinct patterns of humoral immune response.
5. Exploratory correlation-network analysis revealed coordinated, series-specific patterns of taxon–taxon co-variation across the dietary gradients, providing a community-level perspective on microbial reorganization while not implying direct ecological or causal interactions.

Classified reads and relative abundance provided complementary but non-equivalent information, distinguishing representation-consistent from representation-dependent responses. Clustering and exploratory correlation analyses further revealed broader patterns of community organization and co-variation.

Taken together, these findings indicate that the intestinal microbial response to dietary monosaccharides is strongly context-dependent and should be considered in relation to the overall macronutrient composition of the diet rather than to glucose or fructose in isolation.

## Figures and Tables

**Table 1:** Percentage formulation of experimental diets.

	L_6.03_	GL_5.28_ or FL_5.28_	GL_4.70_ or FL_4.70_	GL_4.23_ or FL_4.23_	G_3.85_ or F_3.85_
Casein (%)	15.65	13.71	12.20	10.99	10.00
Milk protein isolate (%)	15.65	13.71	12.20	10.99	10.00
Cellulose (%)	7.83	6.86	6.10	5.50	5.00
Corn oil (%)	7.83	6.86	6.10	5.50	5.00
Lard (%)	45.22	29.71	17.63	7.94	0.00
Glucose or fructose (%)	0.00	22.29	39.66	53.59	65.00
AIN93MX mineral premix (%)	5.48	4.80	4.27	3.85	3.50
AIN93VX vitamin premix (%)	1.57	1.37	1.22	1.10	1.00
Cys, Met, choline (%)	0.78	0.69	0.61	0.55	0.50
Sum (%)	100.00	100.00	100.00	100.00	100.00
Calculated energy contents (kcal/100 g diet)	602.61	528.00	469.83	423.21	385.00
Calculated protein content (g/100 g diet)	31.30	27.43	24.41	21.98	20.00

Letters L, G and F stand for lard, glucose and fructose. Subscripts show the gross energy (GE) content of the diets (kcal/g).

**Table 2:** Daily feed, energy, and nutrient intakes of rats fed the experimental diets.

Parameter	L_6.03_	GL_5.28_	GL_4.70_	GL_4.23_	G_3.85_	FL_5.28_	FL_4.70_	FL_4.23_	F_3.85_
Feed intake	6.35	6.85	7.28	6.57	7.53	7.59	7.74	7.20	7.68
Energy intake *	38.27	36.17	34.20	27.80	28.99	40.08	36.36	30.47	29.57
Glucose/fructose intake	0.00	1.53	2.89	3.52	4.89	1.69	3.07	3.86	4.99
Total lipid intake	3.37	2.51	1.73	0.88	0.38	2.78	1.84	0.97	0.38
Lard intake	2.87	2.04	1.28	0.52	0.00	2.25	1.36	0.57	0.00
Protein intake	1.99	1.88	1.78	1.44	1.51	2.08	1.89	1.58	1.54
Fiber intake	0.50	0.47	0.44	0.36	0.38	0.52	0.47	0.40	0.38

Values are expressed as g/day/100 g BW, except energy intake (* kcal/day/100 g BW). BW, body weight; L_6.03_, lard-rich control; GL, glucose + lard; G, glucose; FL, fructose + lard; F, fructose. Note: Calculated nutrient intakes were derived from the formulated diet composition and the corresponding mean feed intake. Total lipid intake was calculated as the combined intake of lard and corn oil; fiber intake was calculated from the formulated cellulose content. Values are treatment-level calculated exposures and are presented to document the nutritional context of the microbiome comparisons.

**Table 3:** Total sequencing/classified read counts at the phylum and genus levels in the pooled cecal microbiome samples.

**Glucose Dietary Series**	**L_6.03_**	**GL_5.28_**	**GL_4.70_**	**GL_4.23_**	**G_3.85_**
Phylum	49,686	13,920	8866	23,007	62,283
Genus	41,390	10,689	7475	19,643	52,522
**Fructose Dietary Series**	**L_6.03_**	**FL_5.28_**	**FL_4.70_**	**FL_4.23_**	**F_3.85_**
Phylum	49,686	11,893	10,687	8414	41,978
Genus	41,390	9209	8153	6862	37,804

Note: Values represent sequencing/classified read counts from the single pooled cecal microbiome sample for each dietary treatment and should not be interpreted as bacterial abundance. L_6.03_ is the common monosaccharide-free control for both dietary series. Abbreviations: L, lard; GL, glucose + lard; G, glucose; FL, fructose + lard; F, fructose.

## Data Availability

The original contributions presented in the study are included in the article; further inquiries can be directed to the corresponding author.
